# A pilot feeding study for adults with asthma: The healthy eating better breathing trial

**DOI:** 10.1371/journal.pone.0180068

**Published:** 2017-07-13

**Authors:** Emily P. Brigham, Elizabeth C. Matsui, Lawrence J. Appel, Deborah A. Bull, Jean Curtin-Brosnan, Shuyan Zhai, Karen White, Jeanne B. Charleston, Nadia N. Hansel, Gregory B. Diette, Meredith C. McCormack

**Affiliations:** 1 The Johns Hopkins University School of Medicine, Baltimore, Maryland, United States of America; 2 The Johns Hopkins Bloomberg School of Public Health, Baltimore, Maryland, United States of America; Universite de Bretagne Occidentale, FRANCE

## Abstract

**Rationale:**

Evidence from observational studies and to a lesser extent clinical trials suggest that a healthy diet may improve symptoms and lung function in patients with asthma. We conducted a pilot study to determine the feasibility of conducting a larger scale dietary trial and to provide preliminary evidence on the impact of a healthy diet on asthma outcomes.

**Methods:**

In a randomized, two period cross-over trial, participants with asthma received a 4-week dietary intervention followed by a usual diet (or vice versa), separated by a 4-week washout. The dietary intervention was a healthy diet rich in unsaturated fat. During the dietary intervention, participants ate three meals per week on site at the Johns Hopkins ProHealth Research Center. All remaining meals and snacks were provided for participants to consume off-site. During the control diet, participants were instructed to continue their usual dietary intake. Relevant biomarkers and asthma clinical outcomes were assessed at 0, 2, and 4 weeks after starting each arm of the study.

**Results:**

Eleven participants were randomized, and seven completed the full study protocol. Among these seven participants, average age was 42 years, six were female, and six were African American. Participant self-report of dietary intake revealed significant increases in fruit, vegetable, and omega-3 fatty acid intake with the dietary intervention compared to usual diet. Serum carotenoids (eg. lutein and beta-cryptoxanthin) increased in the intervention versus control. Total cholesterol decreased in the intervention versus control diet. There was no consistent effect on asthma outcomes.

**Conclusions:**

The findings suggest that a feeding trial in participants with asthma is feasible. Larger trials are needed to definitively assess the potential benefits of dietary interventions on pulmonary symptoms and function in patients with asthma.

## Introduction

Observational studies have demonstrated harmful effects of the “Western” diet pattern in asthma and conversely a protective effect of healthier diets (such as a “Prudent” or Mediterranean diet pattern) [1–3]. “Western” diet patterns are characterized by high intake of processed meats and fast foods, whereas healthier diets such as the Mediterranean diet are characterized by higher intake of fruits, vegetables, whole grains, lean meats, fish, nuts, and omega-3 fatty acids.

Clinical trials are needed to confirm the effects, if any, of diet on lung symptoms and function in patients with asthma. A limited number of trials explore the effects of dietary counseling on asthma morbidity, with promising results [4–6]. However, no trial to date has investigated the effects of diet on lung symptoms and function using a feeding study design, i.e. providing all meals and snacks to study participants. This approach provides the strongest efficacy data to assess the effects of dietary changes on outcomes. As such, we applied this rigorous study design, as has been done previously to investigate the effect of diet on cardiovascular disease risk factors [7–9].

One of the most widely-recognized feeding studies is the Dietary Approaches to Stop Hypertension (DASH) Trial. A subsequent feeding study, termed the Optimal Macronutrient Intake Trial to Prevent Heart Disease (OmniHeart Trial), tested a DASH-style diet intervention which is rich in unsaturated fat and similar to a Mediterranean diet [7,10]. This diet features high fruit, vegetable, and low-fat dairy intake, with replacement of carbohydrates with olive oil, canola oil, and other monounsaturated fats [11]. The feasibility of testing this diet in adults with asthma is important and cannot be inferred from feeding studies in the general population, given a higher prevalence of food sensitization and food allergy among asthmatics [12,13]. Using a randomized, two period crossover trial of a four week “respiratory healthy” diet intervention versus usual diet as control, we investigated the feasibility of conducting a feeding study in adults with asthma (the Healthy Eating Better Breathing Trial).

## Methods

### Subjects

Adults age 18–50 years with stable asthma were recruited via mailings, flyers in the community, and contacting participants in previous Johns Hopkins asthma studies. Eligibility was determined at a screening visit where informed consent was obtained and criteria included stable, active asthma. Asthma was defined as self-reported physician diagnosis and treatment for asthma within the 12 months prior to enrollment, and stability was defined as no asthma exacerbation (emergency department visit, systemic steroid treatment, or urgent care visit) or respiratory infection in the 4 weeks prior to enrollment. Participants were excluded if they were active smokers, pregnant or breastfeeding, used systemic corticosteroids or warfarin, had another major pulmonary diagnosis or significant systemic illness, reported high alcohol consumption (over 14 drinks per week or six or more drinks on one or more occasions per week), reported food allergy, weighed over 350 pounds or had changed weight by over ten pounds in the two months prior to screening, or reported so few symptoms at screening (Asthma Control Test score of 20 or more) that a change in symptoms was unlikely to be detected. To be eligible, individuals also had to agree to eat at least one meal per day at the study site, three to four days per week for four weeks during the intervention diet.

### Study design

This pilot study used a randomized two period, two treatment crossover design. A four week washout separated the two diets, each of which lasted 4 weeks. A convenience sample of 12 participants was proposed for this feasibility trial. Participants were enrolled by a dedicated study coordinator between September and November of 2012. Participants were randomized to begin with either the control or intervention diet. The random allocation sequence was generated by members of the data core and provided to research staff at the ProHealth facility. During the intervention diet, participants were instructed to consume only foods provided by the study, which included three meals and one snack daily. Food during the intervention diet was prepared and provided by the nutritional staff in the research kitchen at the ProHealth facility. Nutrient targets were based on the “diet rich in unsaturated fat” arm of the OmniHeart Trial, described in detail previously [7,10]. Sample menus can be found in the Online Supplement (Table A in S1 File). Supplied calories were adjusted to achieve stable weight, with anticipated daily caloric needs based on weight and estimated energy expenditure. During the intervention diet, participants ate lunch at the ProHealth facility in Baltimore three to four times per week and received enough for the meals between the in-person visits. Additional meals and snacks were provided to cover unanticipated events (e.g. weather delays) that may have resulted in schedule changes in order to ensure that participants always had access to study food. During the control diet and washout, participants were instructed to consume their typical diet; no meals were provided, and they were not required to visit the ProHealth facility for meals.

### Procedures

Prior to randomization, asthma status was reassessed, and a usual diet assessment was completed via three 24-hour diet recalls. At 0, 2, and 4 weeks of each four-week diet, participants completed the following assessments: Asthma Control Test (ACT) questionnaire [14,15], Asthma-Specific Quality of Life Questionnaire (AQLQ-S) [16–18], Asthma Symptoms Utility Index (ASUI) [19,20], and spirometry and exhaled nitric oxide (eNO) measurements according to ATS guidelines [21,22]. Fasting blood samples were collected at these same timepoints for evaluation of serum carotenoids by high performance liquid chromatography (GENOX Labs) and lipids by spectrophotometry (Quest Diagnostics). To provide further insight into dietary change, participants completed three 24-hour diet recalls during each of the intervention and control diets.

### Outcome measures

Adherence to the dietary intervention was assessed via daily self-reported adherence to the diet, serum carotenoid levels, serum lipids, and 24-hour recalls of dietary intake. Outcomes relevant to asthma morbidity included the ACT, AQLQ, and ASUI questionnaires, spirometry [23], and eNO.

### Statistical analysis

Given sample size and non-normality of sample population data, all summary statistics are presented as medians and interquartile ranges. Biomarkers and asthma clinical outcomes are presented as change at week four (primary analysis) or week two (online supplement) from value obtained at week zero of each respective intervention or control diet. As three 24 hour dietary recalls were performed during each of the screening, intervention diet, and control diet windows, the approach slightly differed; the three recalls were averaged for each week, and changes were presented as differences between the intervention or control diet and the screening week. Individual estimates are graphically represented as change from screening to control and screening to intervention diet. The Wilcoxon signed rank test is used to provide pairwise comparisons of the change in data points across each of the control and intervention diets. A p-value of <0.05 is considered statistically-significant. Statistical analyses were performed using STATA version 13 (College Station, TX) and R 3.0.3 (Vienna, Austria) statistical software.

This trial is registered at clinicaltrials.gov, identifier NCT02904655. The full trial protocol can be accessed through the Johns Hopkins Institutional Review Board or by contacting the corresponding author and is available in the Online Supplement (Appendix A in S1 File). The protocol was approved by the Johns Hopkins Medicine Institutional Review Board (NA_00071879). All participants provided written, informed consent. The data upon which these analyses are based is provided in the Online Supplement (Tables B,C,D,E in S1 File). For participant privacy, given small sample size, data with the potential for participant identification are withheld. For access to an anonymized data set, please contact the corresponding author (MCM). Funding was provided by the National Institute of Environmental Health Sciences and the Environmental Protection Agency.

## Results

**Fig 1:**
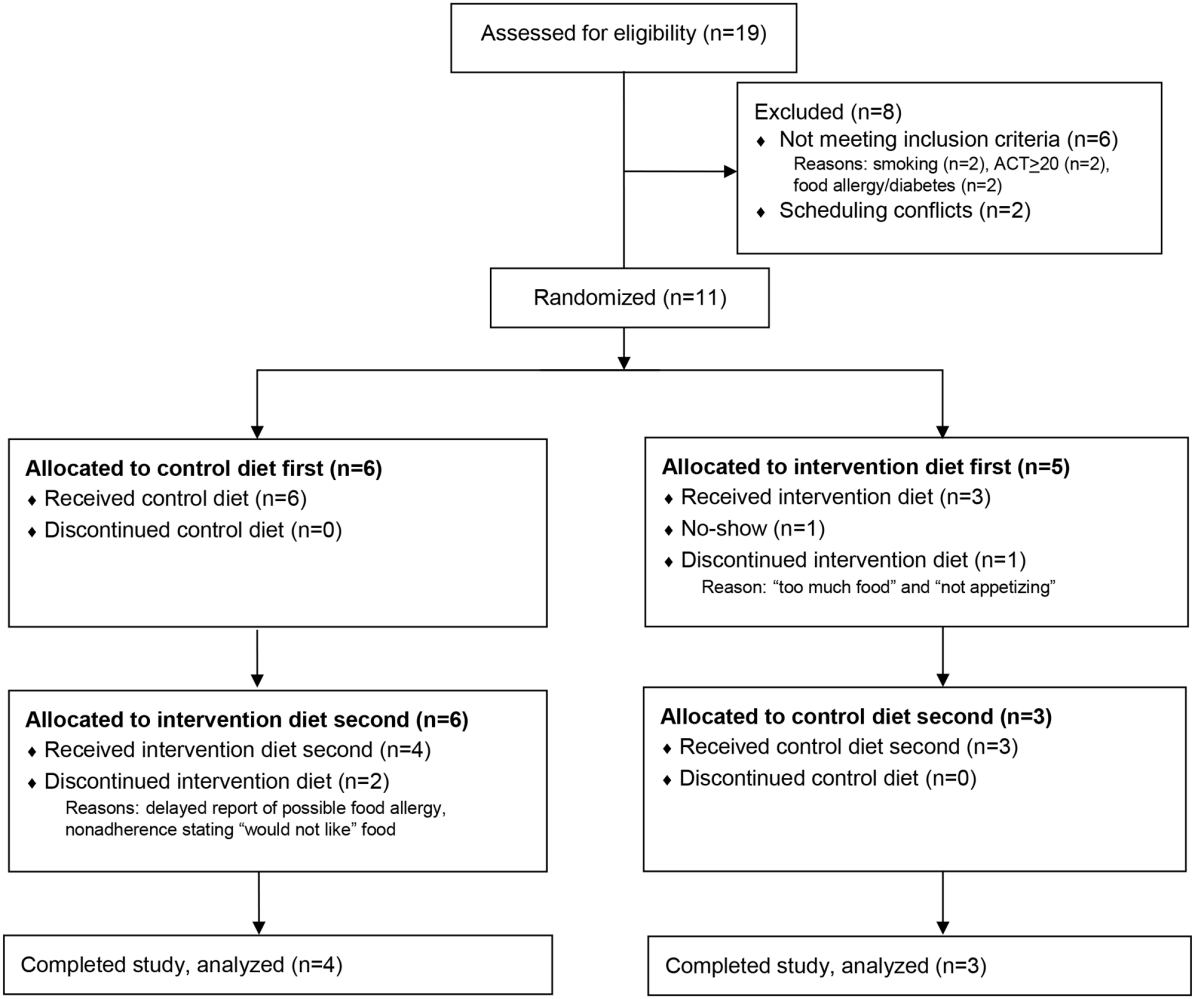
Consort diagram. Participants screened and enrolled.

Nineteen adults were screened and 11 participants were enrolled and randomized. Reasons for screen-failure included active smoking, ACT score of 20 or greater at the time of screening, and inability to commit to the time required for study visits. Four participants discontinued the study after randomization; one prior to initiating the intervention for reasons undisclosed by the participant, and three during the intervention feeding diet. Two participants did not like the meals, and one reported a possible food allergy that was not recalled initially during screening. Seven participants completed the full protocol and their data was used for all analyses (Fig 1).

Participants were predominantly female (6 of 7) and African-American (6 of 7) (Table 1). All participants were obese, with a median body mass index of 38 kg/m^2^, and five reported a low-income.

**Table 1:** Participant characteristics.

Characteristic	n = 7
Age in years	42 (37–50)
Sex, number female	6
Race	
Caucasian	1
Black/African-American	6
Education	
< High school	3
High School	2
Some College	2
Income	
Under $10,000	3
$10,000–49,999	2
>$50,000	2
BMI	38 (33–39)
Ever smoker	2

Absolute numbers and median (25%-75%tile) provided for continuous data; Both ever-smokers reported less than 10 pack-years

**Fig 2:**
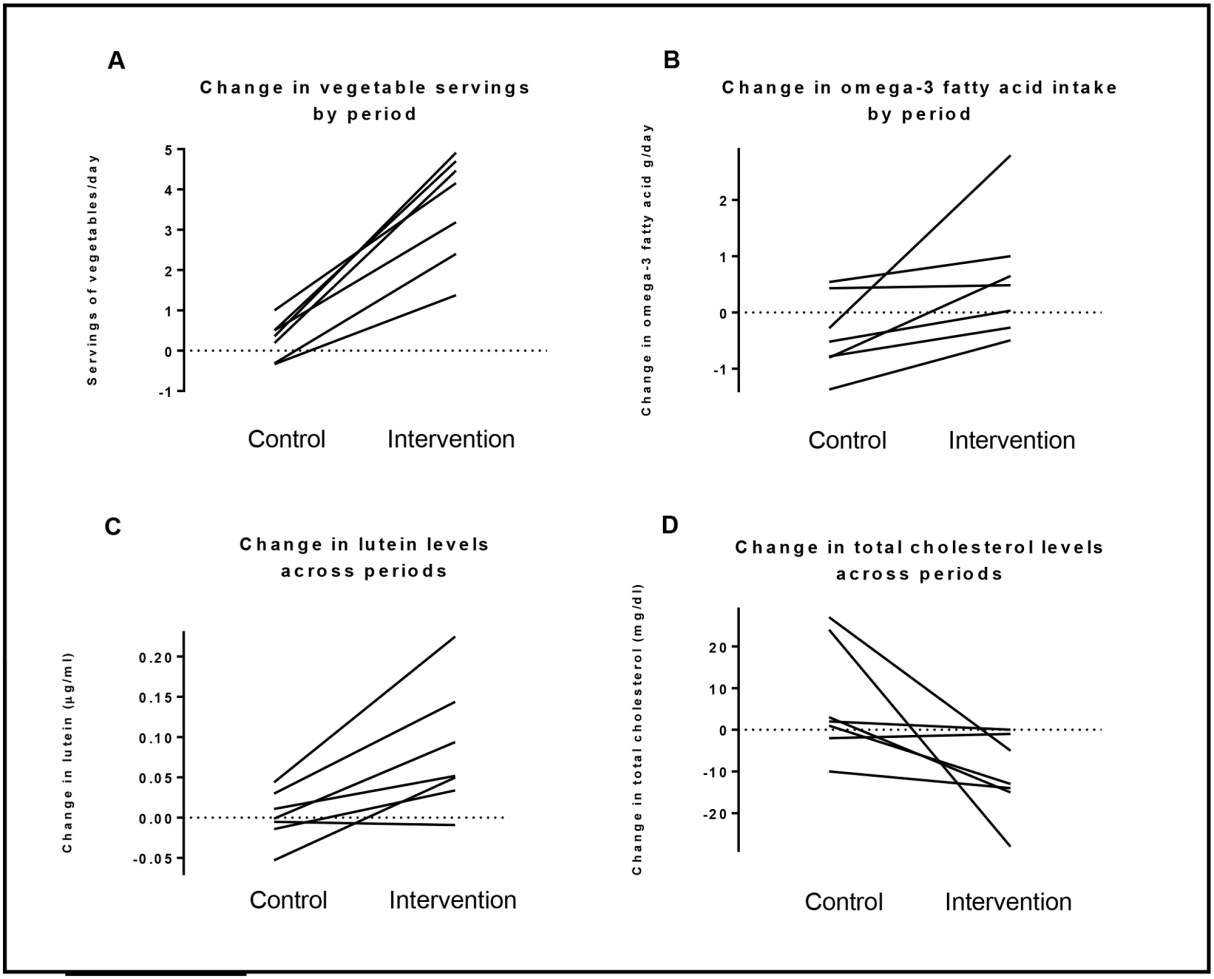
Select adherence outcomes. Data in each graph represent the change in value from week 0 to week 4 of the control diet and the change in value from week 0 to week 4 of the intervention diet. Points are linked by lines connecting responses within individual participants, with the following outcomes represented: (A) vegetable servings; (B) omega-3 fatty acid intake; (C) lutein levels; (D) total cholesterol levels.

Self-report of dietary intake and measurement of biomarkers suggested participant adherence to the dietary intervention. Four out of seven participants denied snacking or meals outside of the study diet. Of the three participants who reported consuming food items outside of the meals provided, this were reported at most on 3 out of the 28 days of the intervention. Participants reported a higher intake of fruits and vegetables during the intervention diet as compared to the control diet, with a median increase of over 2.5 servings of fruit and over 3 servings of vegetables per day (Table 2 and Fig 2A). This increase was corroborated biologically via increases in the several serum carotenoids. For example, lutein and beta-cryptoxanthin increased during the intervention diet as compared to during the control diet at 4 weeks (median lutein -0.001 versus 0.05 μg/ml; median beta-cryptoxanthin 0.001 versus 0.02 μg/ml respectively, Table 3 and Fig 2C). Results were similar at the two and four-week marks (Table F in S1 File).

**Table 2:** Participant-Reported dietary intake from 24 Hour dietary recalls.

	Screening	Control Diet	Intervention Diet	Δ between Diets	P-value
Dietary Intake (servings/day)					
Fruit	0.01	0.79	3.28	2.56	0.02
	(0–0.92)	(0.34–1.25)	(2.25–3.81)	(2.25–2.81)	
Vegetables	1.66	2.16	5.60	3.15	0.02
	(1.16–2.07)	(0.98–3.01)	(3.69–6.53)	(2.68–4.28)	
Whole Grains	0.16	0.88	0.81	0.02	1.00
	(0–1.15)	(0.03–1.27)	(0.53–1.11)	(-1.27–0.53)	
Refined Grains	3.79	3.04	2.28	-0.30	0.40
	(1.56–4.17)	(1.23–4.07)	(1.65–2.74)	(-2.07–1.05)	
Mixed Grains	0	0	0.41	0.33	0.27
	(0–1.01)	(0–0.68)	(0.16–0.52)	(-0.78–0.42)	
Dairy	0.92	0.52	0.95	0.31	0.13
	(0.62–2.72)	(0.43–1.22)	(0.58–2.00)	(0.07–0.78)	
Total Meat	3.07	2.50	2.07	0.54	0.87
	(2.50–4.04)	(0.69–3.47)	(1.76–3.38)	(-1.40–1.18)	
Lean Meat	1.67	0.47	1.86	0.76	0.13
	(0.78–2.36)	(0.02–1.16)	(1.00–3.05)	(-0.33–3.03)	
Processed Meats	0.63	0.08	0	-0.08	0.10
	(0.24–1.57)	(0–1.18)	(0–0)	(-1.18–0)	
Fish/Seafood	1.31	1.00	1.52	0.18	0.27
	(0–3.91)	(0.61–1.61)	(0.61–2.63)	(-0.35–1.47)	
Oil	1.17	0.64	0.67	-0.38	0.34
	(0.78–1.92)	(0.30–2.17)	(0.24–1.64)	(-2.02–0.99)	
Nuts	0	0.64	3.77	1.13	0.18
	(0–0)	(0–3.53)	(1.13–5.68)	(-0.96–5.36)	
Sweets	2.79	1.13	0.93	-0.05	0.40
	(0.51–4.55)	(0.69–3.75)	(0.58–1.08)	(-3.30–0.49)	
Nutrients					
Total fat (%)	31.45	34.39	40.44	3.07	0.06
	(28.30–42.83)	(27.10–40.59)	(35.58–44.70)	(1.80–11.32)	
Saturated (%)	9.73	9.26	7.84	-1.27	0.09
	(8.01–14.57)	(8.63–12.51)	(7.20–11.10)	(-3.05–0.08)	
Monounsaturated (%)	13.56	15.26	18.95	4.28	0.04
	(13.27–19.44)	(11.41–18.00)	(18.23–22.31)	(0.89–8.61)	
Polyunsaturated (%)	6.73	10.75	9.12	1.63	0.06
	(6.01–9.61)	(10.20–13.05)	(6.43–10.79)	(-0.17–6.56)	
Carbohydrate (%)	45.47	49.24	40.00	-3.43	0.24
	(38.47–49.49)	(39.66–50.31)	(38.72–45.49)	(-13.88–0.38)	
Protein (%)	18.66	16.23	17.26	0.14	0.61
	(14.59–23.62)	(15.78–17.31)	(16.39–20.45)	(-0.91–2.68)	
Cholesterol (mg)	338.83	220.90	144.29	-87.68	0.13
	(128.81–366.06)	(126.65–245.40)	(92.48–156.37)	(-131.16–18.36)	
Omega-3 Fatty Acids (g)	1.46	1.36	2.03	0.55	0.02
	(1.03–2.08)	(0.84–1.57)	(1.39–2.72)	(0.46–1.45)	

Data are presented as median (25%-75%tile). Average daily intake computed from mean of 3x 24-hour recalls during each diet. P-value for change between diets.

**Table 3:** Change in serum markers of adherence.

	Baseline	Δ during Control Diet	Δ during Intervention Diet	
Serum Carotenoids (μg/ml)				
Lutein	0.10	-0.001	0.05	0.03
	(0.09–0.13)	(-0.01–0.02)	(0.04–0.12)	
Zeaxanthin	0.04	-0.001	-0.002	0.30
	(0.03–0.05)	(-0.002–0.01)	(-0.004–0.01)	
Lycopene	0.21	-0.02	-0.04	0.38
	(0.11–0.25)	(-0.02–0.02)	(-0.05–0)	
Retinyl palmitate	0.01	0	0.02	0.42
	(0–0.02)	(0–0.004)	(0–0.02)	
Alpha-carotene	0.01	-0.001	0.03	0.24
	(0–0.02)	(-0.006–0.005)	(0.002–0.04)	
Beta-carotene	0.08	0.01	0.06	0.11
	(0.07–0.09)	(-0.01–0.01)	(0.01–0.10)	
Beta-cryptoxanthin	0.03	0.001	0.02	0.03
	(0.03–0.05)	(-0.003–0.006)	(0.01–0.05)	
Retinol	0.46	0.03	-0.03	0.58
	(0.36–0.55)	(-0.01–0.05)	(-0.09–0.02)	
Lipids (mg/dl)				
LDL	79	1	1	0.06
	(64–88)	(0–16)	(-4.5–3)	
HDL	58	-3	-7	0.08
	(53–68)	(-3.5–0)	(-19.5- -5.5)	
Triglycerides	76	2	2	0.94
	(56–92)	(0–16)	(-10.5–11)	
Total cholesterol	153.5	2	-13	0.03
	(142–166)	(-0.5–13.5)	(-14.5- -3)	

Data are presented as median (25%-75%tile). Change from 0 to 4 weeks.

Notable nutrient changes with the intervention included an increase in percentage of intake from monounsaturated fat and decrease in the intake of polyunsaturated fat during the intervention diet as compared to during the control diet (Table 2). Dietary intake, reported by participants in 24-hour recalls during the intervention diet, approximated the composition of the “diet rich in unsaturated fat” arm of the OmniHeart Trial (Table 4). Additionally, participants reported an increase in omega-3-fatty acid intake during the intervention diet as compared to during the control diet (median increase 0.48g versus decrease 0.52g, Table 2 and Fig 2B), consistent with transition to a more Mediterranean-style diet during the intervention.

**Table 4:** Comparison of BREATHE 24-hour recall data to OmniHeart diet composition.

	BREATHE Intervention Diet, Participant-Reported Median Intake	OmniHeart Unsaturated Fat Diet, Goal Intake*
Total fat (%)	40	37
Saturated (%)	8	6
Monounsaturated (%)	19	21
Polyunsaturated (%)	11	10
Carbohydrate (%)	40	48
Protein (%)	17	15
Cholesterol (mg)	144	150

*Reference: (32)

Adherence to the dietary intervention was also supported by changes in measurements in serum cholesterol. A drop in total cholesterol was observed in the study group after 4 weeks of intervention compared to 4 weeks of control (median decrease 13mg/dl versus increase of 2mg/dl, Fig 2D), along with a trend towards decrease in low density lipoprotein levels (Table 4). Median triglyceride levels were not affected. Summary statistics of the change in outcomes of the course of the control and intervention diets were similar at two weeks (online Supplement, S6 Table).

**Fig 3:**
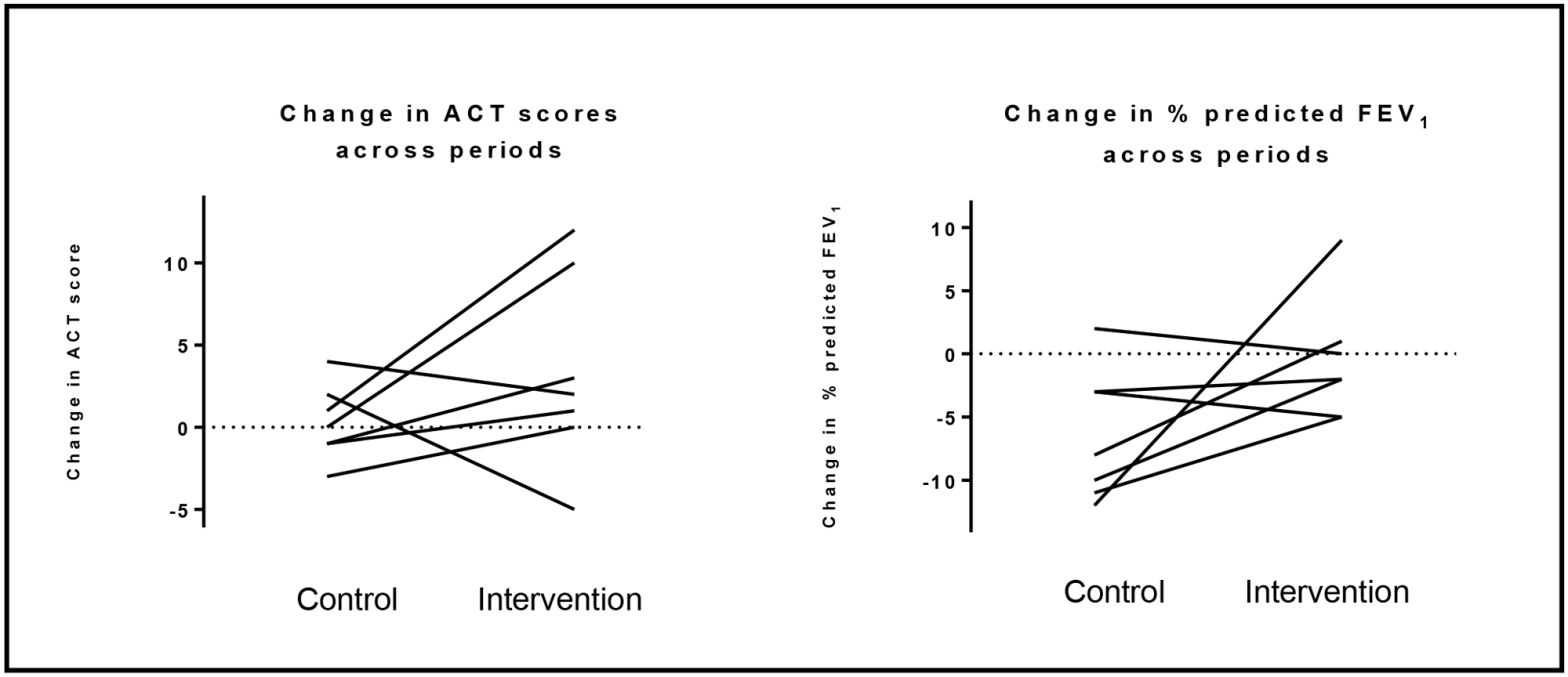
Select asthma morbidity outcomes. Data in each graph represent the change in value from week 0 to week 4 of the control diet and the change in value from week 0 to week 4 of the intervention diet. Points are linked by lines connecting responses within individual participants.

Asthma outcomes assessed using standardized questionnaires, lung function, and FeNO demonstrated variability in response to intervention with modest sample size. Of the outcomes assessed, ACT scores and lung function appeared most responsive to the intervention in the hypothesized direction (Table 5, Fig 3A and 3B). Summary statistics of the change in outcomes of the course of the control and intervention diets were similar at two weeks (Table G in S1 File).

**Table 5:** Change in asthma morbidity outcomes.

	Baseline	Δ during Control Diet	Δ during Intervention Diet	P-value
ACT	20	0	2	0.24
	(18.25–21)	(-1-1.5)	(0.5–6.5)	
ASUI*	0.75	-0.125	-0.5	0.79
	(0.5–1)	(-0.25–0)	(-0.78–0)	
AQLQ^¶^	8.5	-5	-0.5	0.37
	(4.5–13.5)	(-8.5- -1.5)	(-1.25–0.75)	
FEV_1_ (% predicted)	86.5	-8	-2	0.15
	(76.75–97)	(-10.5- -3)	(-3.5–0.5)	
FEV_1_/FVC ratio	0.87	0.005	0.01	0.81
	(0.82–0.89)	(-0.002–0.016)	(0.003–0.03)	
eNO (ppb)	45	-2	2	0.61
	(23–44)	(-4.5–5)	(-12.5–4)	

Data are presented as median (25%-75%tile). ACT: Asthma Control Test (range 5–25, higher score denoting better asthma control); ASUI: Asthma Symptom Utility Index (range 0–1, higher score denoting fewer symptoms); AQLQ: Asthma Quality of Life Questionnaire (range 0–60, higher score denoting worse quality of life); eNO: exhaled nitric oxide, ppb: parts per billion

*n = 6;

^¶^n = 4.

## Discussion

To our knowledge, we report findings from the first feeding study (complete provision of meals) in participants with asthma. The study results suggest that the rigorous deployment of a dietary intervention providing complete meals is feasible in an urban population with asthma, as demonstrated by self-report of dietary change, increases in serum carotenoids and reduction in total cholesterol (biomarkers of adherence) with the intervention. Challenges encountered during the course of the study, including participant enrollment, will be useful to inform a future trial.

While previous studies demonstrate an ability to enact dietary change and suggest a positive effect on asthma outcomes, the Healthy Eating Better Breathing study adds a novel perspective. First, our method of effecting dietary change is unique to the asthma literature. Complete dietary replacement is a highly precise method to alter dietary intake, and though it is labor and cost intensive, it may provide investigators with the highest assurance of participant compliance with the dietary intervention. Second, the majority of our sample was African-American, 5 of 7 were from a low socioeconomic background, and overall subjects demonstrated a median BMI of 38 kg/m^2^. Our findings demonstrate the ability to achieve dietary change in the context of a clinical trial in an urban population that carries a disproportionate burden of asthma prevalence and morbidity [24], who may therefore stand to benefit the most from asthma interventions and are underrepresented in trials to date. Third, the outcomes were assessed at both two and four weeks. Biomarker and metabolic changes were seen as early as two weeks and remained consistent at four weeks. These findings suggest that making measurements as early as two weeks may be a good use of resources in a future study and also demonstrate the ability to sustain the intervention diet for as long as one month using the feeding trial design.

The successes of meal provision as a method of dietary change may be attributable to multiple factors. Participants were financially compensated for their significant time commitment to the trial, including consumption of several meals per week at the facility. All meals were pre-prepared and provided by the research kitchen, simultaneously reducing the burden of food preparation for the participant and eliminating the need for participants to apply knowledge of intervention diet composition to succeed in dietary change. Several participants reported that they had never tasted some of the foods in the intervention diet prior to study enrollment. Had participants been instructed to prepare and consume these foods independently, incomplete understanding of or hesitancy to try preparation techniques could have provided an obstacle to adherence and reduced the potential to achieve nutrient targets. Lastly, participants remarked that they enjoyed eating meals at the clinical research unit with other study subjects, and reported a sense of community with fellow participants in the study.

The modest sample size of subjects completing the study limits the ability to draw conclusions regarding the impact of the healthy diet intervention on asthma health. Limited evidence from other small diet intervention trials, primarily involving dietary counseling with or without vouchers for food purchase, suggested trends in asthma improvement. A study in Australia randomized 79 adults with stable asthma to a counseling intervention of either high or low fruit and vegetable intake. Compliance with the dietary requirements of the intervention, assessed by 24-hour recall, was unmet by 20% of the participants. High intake of fruits and vegetables was associated with a significant increase in serum carotenoids, whereas low intake was associated with a significant drop in serum carotenoids and in FEV_1_ [5]. A second study in New Zealand randomized 35 adults with symptomatic asthma to one of three Mediterranean Diet interventions; over 40 hours of dietary consultation sessions plus provision of olive oil and food vouchers, two hours of dietary consultation plus provision of olive oil and food vouchers, or one dietary consultation, recipes, and free food at the end of the trial [6]. Markers of adherence (Mediterranean Diet Score, serum ascorbic acid, total cholesterol and LDL cholesterol) were influenced favorably with the highest intensity intervention. The study was underpowered to detect clinical endpoints in asthma, though clinically important improvements were seen in AQLQ categories [6]. Lastly, a recent US trial randomized 90 adult participants with uncontrolled asthma to usual care or intense dietary counselling to modify diet to the DASH (Dietary Approaches to Stop Hypertension) Diet [4,8,25]. Odds of reaching goal fruit and vegetable intake in the intervention group, though improved by 1.9 servings per day at 6 months, was not statistically different than the control group. Modest, clinically significant improvements were noted in the Asthma Control Questionnaire [26] and mini-AQLQ [27].

Though a feeding study is unique in the asthma-diet literature and overcomes several potential barriers to diet adherence, this type of study presents several challenges. First, four participants decided to drop-out of the trial after randomization; at least three of these were directly related to food preferences and expected tolerability of the intervention diet. This pilot study, in contrast to the DASH and OmniHeart trials, did not include a pre-randomization, run-in phase, which provides potential participants with firsthand knowledge of the diets and feeding procedures. Either a run-in phase or an intensive discussion of the intervention diet during the consent and enrollment process, including specific menus, ingredients, and quantities of food provided, is therefore suggested to prevent enrollment of individuals at greater risk of drop-out and therefore improve resource allocation in the study. Second, complete meal replacement is expensive and labor intensive. Due to the cost of preparing and delivering the intervention, sample size was limited. However, the use of crossover design increased statistical power as each participant served as an internal control. Participant compensation, an additional cost, is typical for clinical trials but may have positively skewed initial enrollment in the study. Third, though we attempted to enroll participants with stable but symptomatic asthma at baseline, at subsequent baseline exams participants enrolled in the trial, on average, demonstrated well-controlled asthma (ACT median 20). Therefore clinical effects of a dietary intervention may have been too subtle to detect. Fourth, while we provided all meals during the intervention diet with instructions to consume all food and only the food provided, three participants reported several days of nonadherence to provided meals. Still, measurable changes in serum biomarkers, lipids, and non-significant trends in asthma health outcomes were observed.

Notably, the total timeline of this trial was shorter than others published, and may have affected magnitude of effect on clinical outcomes. Two of the previous dietary intervention trials in asthma assess outcomes at the earliest timepoint of three months [6,25], however consistent with our results at two and four weeks, in the 14-week Australian trial of dietary counselling to manipulate fruit and vegetable intake, differences in serum carotenoid values as well as in FEV_1_ and FVC were noted as early as two weeks [5]. Furthermore, trials of dietary change in cardiovascular health have demonstrated a reduction in lipid levels at four weeks comparable to that at six weeks (The OmniHeart Trial, [10]), suggesting that serum metabolic markers reach a new steady state at sometime between zero and four weeks. Additionally, dietary challenge with a high fat meal has produced an effect on lung function as early as 4 hours post intervention, notably with an exaggerated negative effect on lung function among obese asthmatics such as those enrolled in our study [28]. We propose that feeding trials have the potential to provide the highest level of evidence for the effects of a predefined diet on asthma outcomes, and provide a significant opportunity to advance understanding of the effects of diet manipulation on asthma health. There is an express need to build an evidence base for the effect of nutrition on asthma through efficacy studies, both to substantiate a link and refine the diet most effective in improving asthma health. Strong evidence of efficacy provides a foundation for effectiveness and implementation studies that are likewise crucial to this work and move successful interventions to reach a broader population, particularly in areas with limited resources to enact such change. Increasing availability of food delivery and services that focus on and market healthy meals, in addition to targeting facilities where meals are served to attendees, may provide unique opportunities to implement meal replacement on a broader scale.

Feeding trials offer a means to investigate dietary patterns in a highly standardized and rigorous approach, and have been used successfully to study effects of dietary change in other chronic diseases. The findings presented here suggest that a feeding trial in participants with asthma is highly feasible. Given significant questions that remain regarding the effect of dietary pattern change on asthma health, larger trials are needed to evaluate clinical endpoints.

## Supporting information

S1 File **Table A**. Sample Weekly Menu; **Table B**. Data: Serum Markers of Adherence; **Table C**. Data: Asthma Morbidity Outcomes; **Table D**. Data: Reported Dietary Intake in Foods; **Table E**. Data: Reported Dietary Intake in Nutrients; **Table F**. Change in Serum Markers of Adherence at 2 Weeks; **Table G**. Change in Asthma Morbidity Outcomes at 2 Weeks; **Appendix A**. Study Protocol.
(DOCX)

S1 CONSORT Checklist (DOC)
