# Supplementary material for: A pilot feeding study for adults with asthma: The healthy eating better breathing trial
Source: PLoS One. 2017 Jul 13;12(7):e0180068. doi: 10.1371/journal.pone.0180068 (PMC5509132; doi:10.1371/journal.pone.0180068)
Supplement: S1 File — Table A. Sample Weekly Menu; Table B. Data: Serum Markers of Adherence; Table C. Data: Asthma Morbidity Outcomes; Table D. Data: Reported Dietary Intake in Foods; Table E. Data: Reported Dietary Intake in Nutrients; Table F. Change in Serum Markers of Adherence at 2 Weeks; Table G. Change in Asthma Morbidity Outcomes at 2 Weeks; Appendix A. Study Protocol. (DOCX) [file pone.0180068.s001.docx]

**Online Supplement**

**Table A. Sample Weekly Menu**

| **Sunday** | **Monday** | | **Tuesday** | | **Wednesday** | | **Thursday** | **Friday** | | **Saturday** |
| --- | --- | --- | --- | --- | --- | --- | --- | --- | --- | --- |
| Breakfast | Breakfast | | Breakfast | | Breakfast | | Breakfast | Breakfast | | Breakfast |
| Broccoli and Potato Frittata; Watermelon; Bread; Orange Juice; Olive Oil; Margarine | Instant Oatmeal; Yogurt- Light; Pecans; Raisins;  Milk -Skim | Cheerios; Milk – Skim; Almonds; Banana | | Blueberry Waffles; Fresh Strawberries; Almonds; Orange Juice | | Lemon Chickpea Muffin; Pecans; Fresh Orange; Olive Oil; Margarine; Milk - Skim | | Creamy Quinoa Cereal; Almonds; Milk – Skim | | Banana Raspberry Muffin; Yogurt-Light; Pecans; Vegetable Juice; Olive Oil; Margarine |
| Lunch | Lunch | Lunch | | Lunch | | Lunch | | Lunch | | Lunch |
| Chicken Ginger Stir Fry; Unsalted Peanuts; Brown Rice; Fresh Pineapple | Chicken Salad/Whole Wheat Bread; Cherry Tomatoes; Spinach; Mandarin Orange; Salad/Canola Oil Rice Vinegar; Almonds | Tuna Salad; Bread; Asian Slaw with  Oilve Oil; Ambrosia Salad | | White Chicken; Corn Chili; Greek Vegetable Salad/Herb Vinegrette; Broccoli; 2% Cottage Cheese; Peaches | | Greek Tuna Pockets; Feta Cheese; Broccoli Salad with Olive Oil; Watermelon | | Provolone Grilled Cheese Sandwich- with Olive Oil; Tomato Basil Soup; Cole Slaw; Banana | | Herbed Beef Pasta; Mozzarella Cheese-reduced fat; Celery/Carrot Sticks; Fresh Orange |
| Dinner | Dinner | Dinner | | Dinner | | Dinner | | | Dinner | Dinner |
| Pork; Yams; Collards with Olive Oil; Olive Oil; Margarine | Cozy Spiced Beef; Broccoli; Ginger Peach Melba | Mesquite Lime Chicken; Stewed Tomatoes; Black Olives; Turnip Greens With Olive Oil; Pears | | Meatloaf; Mashed Potatoes; Kale with Olive Oil; Olive Oil; Margarine; Cantaloupe | | Rosemary Baked Chicken; Mushroom Barley Pilaf; Cooked Spinach; Apricot Halves | | | Creamy Horseradish Salmon; Black Bean, Mango/Citrus Salsa; Ginger Orange Carrot | Baked Cod – Panko Crumbs and Olive Oil; Brown Rice; Spiced Roasted Brussels Sprouts; Dried Apricots |
| Snack | Snack | Snack | | Snack | | Snack | | | Snack | Snack |
| Unsweetened Applesauce; Wheat Crackers; Peanut Butter; Milk - Skim | Cheddar Cheese – reduced fat; Carrots; Celery; Orange Juice | Raisins; Unsalted Peanuts; Orange Juice | | Pears; Carrot-Spice Muffins; Olive Oil; Margarine; Milk-Skim | | Strawberry Salsa; Cinnamon Tortilla Chips; Unsalted Peanuts | | | Yogurt-Light; Cantaloupe | Prunes; Unsalted Peanuts; Milk - Skim |

| **Table B. Data: Serum Markers of Adherence** | | | | | | | | |  |  |  |  |  |  |
| --- | --- | --- | --- | --- | --- | --- | --- | --- | --- | --- | --- | --- | --- | --- |
|  |  |  | Serum Carotenoids (µg/ml) | | | | | | | | Serum Lipids (mg/ml) | | | |
| Participant | Week | C/I | lutein | zeaxanthin | lycopene | retinyl palmitate | α-carotene | β-carotene | β-cryptoxanthin | retinol | LDL | HDL | triglycerides | TC |
| 1 | 0 | I | 0.06 | 0.026 | 0.253 | 1.00E-04 | 0.0017 | 0.086 | 0.028 | 0.329 | 82 | 45 | 77 | 142 |
|  | 2 | I | 0.073 | 0.031 | 0.162 | 1.00E-04 | 1.00E-04 | 0.099 | 0.032 | 0.333 | 81 | 44 | 74 | 140 |
|  | 4 | I | 0.051 | 0.022 | 0.084 | 0.022 | 1.00E-04 | 0.076 | 0.028 | 0.173 | 85 | 26 | 90 | 129 |
|  | 0 | C | 0.087 | 0.043 | 0.212 | 1.00E-04 | 0.002 | 0.085 | 0.03 | 0.328 | 77 | 53 | 56 | 141 |
|  | 2 | C | 0.118 | 0.059 | 0.34 | 1.00E-04 | 0.011 | 0.108 | 0.049 | 0.55 | 80 | 55 | 70 | 149 |
|  | 4 | C | 0.082 | 0.042 | 0.188 | 1.00E-04 | 0.001 | 0.093 | 0.037 | 0.388 | 78 | 52 | 60 | 142 |
| 2 | 0 | C | 0.048 | 0.019 | 0.027 | 0.022 | 1.00E-04 | 0.071 | 0.008 | 0.411 | 47 | 53 | 68 | 114 |
|  | 2 | C | 0.077 | 0.017 | 0.025 | 0.034 | 1.00E-04 | 0.089 | 0.013 | 0.476 | 47 | 54 | 76 | 116 |
|  | 4 | C | 0.059 | 0.016 | 0.038 | 0.022 | 0.0096 | 0.075 | 0.0096 | 0.406 | 48 | 50 | 70 | 112 |
|  | 0 | I | 0.052 | 0.016 | 0.092 | 1.00E-04 | 0.002 | 0.078 | 0.014 | 0.419 | 56 | 48 | 52 | 114 |
|  | 2 | I | 0.135 | 0.024 | 0.068 | 1.00E-04 | 0.004 | 0.095 | 0.017 | 0.44 | 58 | 44 | 48 | 112 |
|  | 4 | I | 0.104 | 0.02 | 0.056 | 1.00E-04 | 0.007 | 0.102 | 0.027 | 0.425 | 57 | 44 | 61 | 113 |
| 3 | 0 | I | 0.09 | 0.037 | 0.212 | 1.00E-04 | 0.002 | 0.065 | 0.026 | 0.349 | 58 | 71 | 75 | 144 |
|  | 2 | I | 0.237 | 0.047 | 0.151 | 0.021 | 0.017 | 0.15 | 0.086 | 0.396 | 50 | 70 | 86 | 137 |
|  | 4 | I | 0.234 | 0.06 | 0.232 | 1.00E-04 | 0.05 | 0.203 | 0.099 | 0.322 | 61 | 71 | 62 | 144 |
|  | 0 | C | 0.147 | 0.07 | 0.25 | 0.021 | 0.009 | 0.075 | 0.047 | 0.473 | 61 | 70 | 81 | 147 |
|  | 2 | C | 0.216 | 0.101 | 0.461 | 0.019 | 0.022 | 0.115 | 0.054 | 0.557 | 70 | 53 | 66 | 136 |
|  | 4 | C | 0.177 | 0.074 | 0.358 | 0.021 | 0.01 | 0.112 | 0.047 | 0.617 | 71 | 62 | 80 | 149 |
| 4 | 0 | C | 0.1 | 0.042 | 0.3 | 0.02 | 0.009 | 0.07 | 0.096 | 0.641 | 81 | 60 | 96 | 160 |
|  | 2 | C | 0.119 | 0.046 | 0.231 | 0.017 | 0.005 | 0.065 | 0.082 | 0.566 | 86 | 56 | 91 | 160 |
|  | 4 | C | 0.144 | 0.061 | 0.323 | 0.028 | 0.004 | 0.077 | 0.086 | 0.623 | 80 | 57 | 128 | 163 |
|  | 0 | I | 0.13 | 0.052 | 0.203 | 0.025 | 0.066 | 0.123 | 0.086 | 0.556 | 94 | 61 | 79 | 171 |
|  | 2 | I | 0.337 | 0.072 | 0.319 | 0.06 | 0.066 | 0.157 | 0.088 | 0.666 | 75 | 53 | 125 | 153 |
|  | 4 | I | 0.355 | 0.092 | 0.184 | 1.00E-04 | 0.034 | 0.103 | 0.084 | 0.764 | 88 | 54 | 71 | 156 |
| 5 | 0 | C | 0.105 | 0.038 | 0.318 | 0.021 | 0.021 | 0.18 | 0.049 | 0.293 | 99 | 77 | 37 | 183 |
|  | 2 | C | 0.101 | 0.046 | 0.285 | 0.021 | 0.016 | 0.19 | 0.136 | 0.335 | 123 | 81 | 39 | 212 |
|  | 4 | C | 0.091 | 0.036 | 0.212 | 0.028 | 0.015 | 0.148 | 0.071 | 0.325 | 121 | 78 | 38 | 207 |
|  | 0 | I | 0.119 | 0.047 | 0.335 | 1.00E-04 | 0.03 | 0.232 | 0.065 | 0.445 | 127 | 88 | 44 | 224 |
|  | 2 | I | 0.188 | 0.051 | 0.287 | 1.00E-04 | 0.058 | 0.374 | 0.101 | 0.358 | 128 | 68 | 55 | 207 |
|  | 4 | I | 0.153 | 0.039 | 0.275 | 0.022 | 0.063 | 0.332 | 0.115 | 0.489 | 124 | 63 | 46 | 196 |
| 6 | 0 | I | 0.089 | 0.03 | 0.147 | 1.00E-04 | 0.024 | 0.082 | 0.013 | 0.551 | 89 | 58 | 96 | 166 |
|  | 2 | I | 0.218 | 0.037 | 0.135 | 0.03 | 0.054 | 0.193 | 0.063 | 0.595 | 101 | 45 | 114 | 169 |
|  | 4 | I | 0.139 | 0.027 | 0.109 | 0.02 | 0.062 | 0.178 | 0.034 | 0.465 | 96 | 51 | 71 | 161 |
|  | 0 | C | 0.145 | 0.049 | 0.09 | 0.023 | 0.016 | 0.095 | 0.027 | 0.669 | 74 | 58 | 57 | 143 |
|  | 2 | C | 0.124 | 0.038 | 0.071 | 1.00E-04 | 0.029 | 0.14 | 0.021 | 0.629 | 91 | 60 | 59 | 163 |
|  | 4 | C | 0.092 | 0.03 | 0.07 | 1.00E-04 | 0.027 | 0.104 | 0.02 | 0.626 | 97 | 62 | 54 | 170 |
| 7 | 0 | C | 0.12 | 0.034 | 0.124 | 1.00E-04 | 0.026 | 0.077 | 0.03 | 0.511 | 84 | 53 | 144 | 166 |
|  | 2 | C | 0.119 | 0.04 | 0.15 | 1.00E-04 | 0.021 | 0.063 | 0.034 | 0.519 | 82 | 56 | 165 | 171 |
|  | 4 | C | 0.119 | 0.042 | 0.107 | 1.00E-04 | 0.02 | 0.056 | 0.035 | 0.542 | 73 | 49 | 172 | 156 |
|  | 0 | I | 0.145 | 0.044 | 0.106 | 0.022 | 0.017 | 0.072 | 0.045 | 0.608 | 75 | 53 | 160 | 160 |
|  | 2 | I | 0.315 | 0.059 | 0.107 | 0.037 | 0.029 | 0.126 | 0.087 | 0.715 | 56 | 40 | 184 | 133 |
|  | 4 | I | 0.239 | 0.042 | 0.148 | 0.04 | 0.044 | 0.136 | 0.096 | 0.511 | 47 | 33 | 331 | 146 |
|  | | | | | | | | |  |  |  |  |  |  |

Serum carotenoids and lipids by participant, week of measurement, and during control (C) or intervention (I) diets. Order of C or I is randomization order.

| **Table C. Data: Asthma Morbidity Outcomes** | | | | | | | | |
| --- | --- | --- | --- | --- | --- | --- | --- | --- |
|  | | | Asthma Morbidity Outcomes | | | | | |
| Participant | Week | C/I | ACT | ASUI | AQLQ | FEV_1_ (% predicted) | FEV_1_/FVC ratio | eNO (ppb) |
| 1 | 0 | I | 14 | 0 | 0 | 101 | 0.7769231 | 8 |
|  | 2 | I | 25 | 0.25 | 0 | 96 | 0.7741935 | 10 |
|  | 4 | I | 24 | 0 | 0 | 101 | 0.7829457 | 15 |
|  | 0 | C | 23 | 0 | 0 | 93 | 0.775 | 8 |
|  | 2 | C | 24 | 0 | 0 | 102 | 0.7846154 | 9 |
|  | 4 | C | 23 | 0 | 0 | 95 | 0.7723577 | 6 |
| 2 | 0 | C | 16 | . | . | 73 | 0.8588235 | 28 |
|  | 2 | C | 19 | 0.5 | 12 | 78 | 0.975 | 19 |
|  | 4 | C | 20 | 0.5 | 10 | 62 | 0.984127 | 23 |
|  | 0 | I | 18 | 1.5 | 9 | 80 | 0.9876543 | 40 |
|  | 2 | I | 18 | 1.25 | 4 | 93 | 1.0108696 | 25 |
|  | 4 | I | 20 | 0.25 | 4 | 75 | 0.9868421 | 17 |
| 3 | 0 | I | 19 | 1.25 | 15 | 85 | 0.8762887 | 88 |
|  | 2 | I | 13 | 0.5 | 33 | 103 | 0.8728814 | 90 |
|  | 4 | I | 14 | 0.75 | 18 | 83 | 0.7904762 | 74 |
|  | 0 | C | 19 | 0.25 | 13 | 97 | 0.8738739 | 28 |
|  | 2 | C | 21 | 0.25 | 12 | 88 | 0.8073395 | 36 |
|  | 4 | C | 21 | 0 | 5 | 87 | 0.8365385 | 34 |
| 4 | 0 | C | 21 | 0.75 | 9 | 99 | 0.8918919 | 131 |
|  | 2 | C | 21 | 0.75 | 9 | 98 | 0.8828829 | 87 |
|  | 4 | C | 20 | 0.75 | 7 | 96 | 0.8971963 | 95 |
|  | 0 | I | 21 | 0.75 | 8 | 102 | 0.9189189 | 109 |
|  | 2 | I | 23 | 0.25 | 7 | 101 | 0.9181818 | 71 |
|  | 4 | I | 24 | 0 | 6 | 97 | 0.9326923 | 114 |
| 5 | 0 | C | 20 | 1 | . | 61 | 0.7625 | 33 |
|  | 2 | C | 16 | 1 | 24 | 54 | 0.7605634 | 17 |
|  | 4 | C | 17 | 0.5 | 19 | 58 | 0.7733333 | 37 |
|  | 0 | I | 20 | 0.5 | 24 | 76 | 0.8085106 | 34 |
|  | 2 | I | 21 | 1 | 16 | 76 | 0.8351648 | 27 |
|  | 4 | I | 20 | 1 | 10 | 74 | 0.8314607 | 23 |
| 6 | 0 | I | 12 | 2 | 22 | 73 | 0.8690476 | 21 |
|  | 2 | I | 21 | 0.5 | 14 | 79 | 1.0821918 | 28 |
|  | 4 | I | 24 | 1 | . | 82 | 0.9647059 | 23 |
|  | 0 | C | 23 | 0.5 | 0 | 79 | 1.025974 | 20 |
|  | 2 | C | 24 | 0.25 | 1 | 76 | 1.0555556 | 13 |
|  | 4 | C | 24 | 0.5 | 2 | 67 | 1.046875 | 16 |
| 7 | 0 | C | 23 | 0.5 | 16 | 97 | 0.8660714 | 45 |
|  | 2 | C | 22 | 0.5 | 7 | 91 | 0.8584906 | 58 |
|  | 4 | C | 22 | 0.25 | 6 | 89 | 0.8640777 | 57 |
|  | 0 | I | 21 | 0.75 | 6 | 88 | 0.8543689 | 36 |
|  | 2 | I | 25 | 0.5 | 2 | 90 | 0.9 | 59 |
|  | 4 | I | 22 | 0.75 | 5 | 89 | 0.89 | 39 |
|  | | | | | | | | |

Asthma morbidity outcomes by participant, week of measurement, and during control (C) or intervention (I) diets. Order of C or I is randomization order.

| **Table D. Data: Reported Dietary Intake in Foods** | | | | | | | | |  | |  | |  | |  | |  | |  | |  |
| --- | --- | --- | --- | --- | --- | --- | --- | --- | --- | --- | --- | --- | --- | --- | --- | --- | --- | --- | --- | --- | --- |
|  | | Dietary Intake (servings/day) | | | | | | | | | | | | | | | | | | | |
| Participant | Time | fruit | vegetables | whole grains | refined grains | mixed grains | dairy | total meat | | lean meat | | processed meats | | fish/  seafood | | oil | | nuts | | sweets | |
| 1 | S | 1.263333 | 1.160667 | 1.727667 | .417 | 0 | .4243333 | 0 | | 0 | | 0 | | 3.905333 | | 0 | | 0 | | 0 | |
|  | I | 2.226333 | 2.538 | 1.073667 | .614 | 1 | .5103333 | 1.756 | | 1 | | 0 | | 1.258667 | | .6666667 | | .6666667 | | .8623333 | |
|  | C | 1.590333 | .8273333 | 1.207333 | .5283334 | .6666667 | .4423333 | .5786666 | | .2423333 | | .0783333 | | 1.613 | | 0 | | 0 | | .032 | |
| 2 | S | 0 | 1.291333 | 0 | 9.319667 | 0 | 2.720333 | 4.013 | | 1.668333 | | 1.566333 | | 0 | | .976 | | 0 | | 11.534 | |
|  | I | 2.253667 | 3.694333 | .529 | 2.000667 | .2186667 | .581 | 1.693333 | | .4536667 | | 0 | | 1.517333 | | 3 | | 1.128667 | | .448 | |
|  | C | 0 | .977 | 0 | 4.068 | 1 | 1.222333 | 2.500667 | | 1.160333 | | .6816667 | | 0 | | .2986667 | | 0 | | 3.75 | |
| 3 | S | .0063333 | 2.002333 | 0 | 1.888333 | 0 | .9916667 | 2.496333 | | 2.257667 | | .2386667 | | 2.591 | | .78 | | 0 | | 2.794667 | |
|  | I | 3.142667 | 6.16 | 1.105333 | 1.654 | 0 | 3.129 | 4.014667 | | 3.245333 | | 0 | | .6113333 | | .6666667 | | 5.998667 | | .5786666 | |
|  | C | .3353333 | 3.006 | .8836667 | 4.364333 | 0 | .1246667 | 11.16833 | | 0 | | 3.298333 | | .6113333 | | 1.836333 | | .635 | | 6.536667 | |
| 4 | S | .9166667 | .8966666 | .908 | 1.562667 | 1.007667 | .9216667 | 4.044667 | | .9103333 | | 2.545667 | | 0 | | 1.167667 | | 0 | | .514 | |
|  | I | 3.807667 | 5.599667 | 0 | 2.282333 | .1646667 | 1.996333 | 3.376667 | | 3.05 | | .0463333 | | .5173333 | | .2423333 | | 5.073 | | 1.078 | |
|  | C | 1.25 | 1.402667 | 1.268333 | 1.232 | 0 | 1.219 | 2.834667 | | .0246667 | | 0 | | 1 | | .6236667 | | 3.527333 | | 1 | |
| 5 | S | 0 | 2.067667 | 0 | 3.789 | .8926667 | .8613333 | 2.945333 | | 2.357333 | | .335 | | 4.702 | | 1.414 | | 0 | | .851 | |
|  | I | 4.379333 | 6.535333 | .5516667 | 3.318667 | .411 | .9533334 | 2.074667 | | 1.858 | | 0 | | 2.034667 | | 1.635333 | | 3.770333 | | 1.177333 | |
|  | C | .643 | 2.258 | .5333334 | 1.368667 | 0 | .516 | 3.47 | | 2.192333 | | 1.175667 | | .7173333 | | .6446667 | | 5.15 | | .686 | |
| 6 | S | 0 | 2.790333 | 1.149333 | 4.166 | 0 | .6226667 | 10.90967 | | 4.379 | | .9173334 | | 0 | | 3.781 | | 1.763667 | | 4.208333 | |
|  | I | 3.664 | 7.700334 | .806 | 2.500333 | .4213333 | .7403333 | 2.058 | | 1.011333 | | 0 | | 3.273 | | 0 | | 2.568667 | | 1.079 | |
|  | C | .883 | 3.149333 | 2.333333 | 3.7 | 0 | .4343333 | .682 | | .682 | | 0 | | 3.091667 | | 2.166667 | | 3.527333 | | 1.128667 | |
| 7 | S | .3333333 | 1.655333 | .159 | 3.844 | 2.141 | 2.911 | 3.074 | | .7766667 | | .6276667 | | 1.313 | | 1.922 | | 0 | | 4.552667 | |
|  | I | 3.288333 | 4.842333 | 1.552 | 2.739 | .5186667 | 1.462 | 2.557667 | | 1.884 | | 0 | | 2.633667 | | .6666667 | | 5.677 | | .9313333 | |
|  | C | .7853333 | 2.160667 | .0286667 | 3.039667 | 0 | 1.188333 | 1.458 | | .4703333 | | 0 | | 1.159667 | | 2.683667 | | 0 | | 1.808667 | |

Reported dietary intake by participant during screening (S), control (C), or intervention (I) diets. Each data point represents mean of 3x24 hour recalls during that time diet.

| **Table E. Data: Reported Dietary Intake in Nutrients** | | | | | | |  |  |  |  |
| --- | --- | --- | --- | --- | --- | --- | --- | --- | --- | --- |
|  | | Nutrients | | | | | | | | |
| Participant |  | Total fat (%) | Saturated fat (%) | Monounsaturated fat (%) | Polyunsaturated fat (%) | Carbohydrate (%) | | Protein (%) | Cholesterol (mg/day) | Omega-3 fatty acids (g/day) |
| 1 | S | 21.806 | 7.338667 | 9.083667 | 5.383667 | 45.46533 | | 32.68333 | 82.96934 | 1.461 |
|  | I | 28.739 | 7.846333 | 12.19933 | 8.693334 | 50.697 | | 20.447 | 92.47567 | 1.192333 |
|  | C | 24.81533 | 8.199667 | 11.312 | 5.303667 | 50.31267 | | 24.86533 | 56.61667 | 0.680333 |
| 2 | S | 28.47067 | 8.993667 | 13.467 | 6.01 | 56.949 | | 14.528 | 380.609 | 1.361333 |
|  | I | 44.697 | 9.338 | 22.30733 | 13.05167 | 39.99833 | | 15.28567 | 89.75367 | 1.393 |
|  | C | 27.102 | 9.260333 | 11.41333 | 6.428333 | 57.103 | | 15.78233 | 220.8993 | 0.839667 |
| 3 | S | 28.30333 | 8.009667 | 13.56433 | 6.729333 | 49.48567 | | 22.17433 | 128.8057 | 1.027333 |
|  | I | 43.97767 | 11.235 | 22.28067 | 10.462 | 38.71833 | | 17.26233 | 157.539 | 2.026333 |
|  | C | 41.805 | 14.285 | 18.00433 | 9.515667 | 42.14967 | | 14.67133 | 436.1203 | 1.567667 |
| 4 | S | 42.82667 | 14.57 | 19.805 | 8.451667 | 38.46567 | | 18.662 | 366.061 | 1.011333 |
|  | I | 45.71567 | 11.09867 | 23.867 | 10.75 | 35.36133 | | 18.91233 | 156.37 | 1.496 |
|  | C | 34.39267 | 10.012 | 15.26133 | 9.119333 | 49.23667 | | 16.22967 | 162.188 | 1.439667 |
| 5 | S | 45.372 | 15.26633 | 19.43767 | 10.668 | 31.02733 | | 23.622 | 285.666 | 2.075667 |
|  | I | 38.08767 | 7.363667 | 18.22633 | 12.49767 | 45.48733 | | 16.43067 | 115.0423 | 2.723 |
|  | C | 40.59 | 8.631667 | 19.292 | 12.66633 | 30.454 | | 16.28667 | 245.398 | 1.274 |
| 6 | S | 35.39633 | 9.734334 | 16.053 | 9.609 | 45.81033 | | 18.64067 | 356.56 | 2.725333 |
|  | I | 35.581 | 6.967333 | 18.417 | 10.19667 | 42.85367 | | 21.61167 | 145.0087 | 2.230667 |
|  | C | 33.778 | 8.847 | 14.13633 | 10.79467 | 50.2 | | 15.99533 | 126.6497 | 1.355 |
| 7 | S | 31.453 | 11.537 | 13.27 | 6.646 | 43.69067 | | 14.59033 | 338.829 | 1.914333 |
|  | I | 40.44667 | 7.204334 | 18.95 | 14.29233 | 39.038 | | 16.39467 | 144.292 | 4.703667 |
|  | C | 37.381 | 12.51233 | 17.13767 | 7.731 | 39.65567 | | 17.30567 | 231.9733 | 1.633667 |

Reported dietary intake by participant during screening (S), control (C), or intervention (I) diets. Each data point represents mean of 3x24 hour recalls during that time diet.

| **Table F. Change in Serum Markers of Adherence at 2 Weeks** | | | | |
| --- | --- | --- | --- | --- |
|  | Baseline | Δ during Control Diet from Baseline | Δ during Intervention Diet from Baseline |  |
| Serum Carotenoids (µg/ml) | | | | |
| Lutein | 0.10 | 0.02 | 0.13 | 0.03 |
|  | (0.09-0.13) | (-0.003-0.03) | (0.08-0.16) |  |
| Zeaxanthin | 0.04 | 0.01 | 0.008 | 0.81 |
|  | (0.03-0.05) | (0.001-0.01) | (0.006-0.01) |  |
| Lycopene | 0.21 | -0.002 | -0.02 | 0.21 |
|  | (0.11-0.25) | (-0.03-0.08) | (-0.05- -0.006) |  |
| Retinyl palmitate | 0.01 | 0 | 0.02 | 0.11 |
|  | (0-0.02) | (-0.003-0) | (0-0.03) |  |
| Alpha-carotene | 0.01 | 0 | 0.01 | 0.11 |
|  | (0-0.02) | (-0.005-0.01) | (0.001-0.02) |  |
| Beta-carotene | 0.08 | 0.02 | 0.05 | 0.08 |
|  | (0.07-0.09) | (0.003-0.03) | (0.03-0.10) |  |
| Beta-cryptoxanthin | 0.03 | 0.005 | 0.04 | 0.38 |
|  | (0.03-0.05) | (-0.001-0.01) | (0.004-0.05) |  |
| Retinol | 0.46 | 0.04 | 0.04 | 0.94 |
|  | (0.36-0.55) | (-0.02-0.07) | (0.01-0.08) |  |
| Lipids (mg/dl) | | | | |
| LDL | 79 | 5 | -1 | 0.03 |
|  | (64-88) | (1.5-13) | (-13.5-1.5) |  |
| HDL | 58 | 2 | -8 | 0.18 |
|  | (53-68) | (-1.5-2.5) | (-13- -2.5) |  |
| Triglycerides | 76 | 2 | 11 | 0.38 |
|  | (56-92) | (-1.5-11) | (4 - 21) |  |
| Total cholesterol | 153.5 | 5 | -7 | 0.04 |
|  | (142-166) | (1-14) | (-17.5 - -2) |  |
| Data are presented as median (25%-75%tile). | | | | |

| **Table G. Change in Asthma Morbidity Outcomes at 2 Weeks** | | | | |
| --- | --- | --- | --- | --- |
|  | Baseline | Δ during Control Diet from Baseline | Δ during Intervention Diet from Baseline | P-value |
| ACT | 20 | 1 | 2 | 0.31 |
|  | (18.25-21) | (-1-2) | (0.5-6.5) |  |
| ASUI^*^ | 0.63 | 0 | -0.38 | 0.29 |
|  | (0.44-0.81) | (0-0) | (-0.69-0.13) |  |
| AQLQ^$^ | 8.5 | 0 | -1 | 0.86 |
|  | (1.5-14.5) | (-1-0) | (-4-0) |  |
| FEV_1_ (% predicted) | 86.5 | -3 | 2 | 0.29 |
|  | (76.75-97) | (-6.5-2) | (-0.5-9.5) |  |
| FEV_1_/FVC ratio | 0.87 | -0.002 | 0.02 | 0.38 |
|  | (0.82-0.89) | (-0.008-0.02) | (-0.002-0.04) |  |
| eNO (ppb) | 45 | -7 | 2 | 0.20 |
|  | (23-44) | (-12.5-4.5) | (-11-4.5) |  |

Data are presented as median (25%-75%tile). ACT: Asthma Control Test (range 5-25, higher score denoting better asthma control); ASUI: Asthma Symptom Utility Index (range 0-1, higher score denoting fewer symptoms); AQLQ: Asthma Quality of Life Questionnaire (range 0-60, higher score denoting worse quality of life); eNO: exhaled nitric oxide, ppb: parts per billion ^*^n=6; ^$^n=5.

**Appendix A. Study Protocol**

**Dietary Interventions in Asthma Treatment: Healthy Eating Better Breathing Asthma Diet Pilot Study**

1. **Abstract**

**Background:** A Mediterranean-like diet (MED DIET) has been shown to improve the risk profile of an inflammatory disease, cardiovascular disease, and so may have similar effects on another inflammatory disease, asthma.

**Objectives:** The objective of the study is to demonstrate feasibility of a randomized, controlled clinical trial of a dietary intervention in a population of adults with asthma in preparation for a large definitive trial aimed at improving asthma clinical outcomes and improving markers of inflammation and oxidative stress.

**Methods:** The study is a pilot study of a randomized crossover trial to assess the feasibility of doing a randomized crossover and to understand variances of clinical and inflammatory outcomes to inform the design of a larger trial. Ten to twelve adults (ages 18-50) who meet eligibility criteria will be randomized to either the MED DIET or a control diet, the participant’s usual diet (control). At baseline, FENO, FEV_1_, nasal epithelial gene expression, symptoms, and biomarkers of oxidative stress and inflammation in urine, nasal lavage fluid, and serum will be assessed. Participants will receive either the MED DIET or will continue with their regular diet for 4 weeks. They will be randomized into 1 of 2 sequences, and will then have assessment of outcomes (FENO, FEV_1_, symptoms, nasal epithelial gene expression, and biomarkers of oxidative stress and inflammation in urine, nasal lavage fluid, and serum) again at 2 weeks and at 4 weeks after starting the diet. The participants will then have a 2-8 week washout period and then have repeat assessment of FENO, FEV_1_, symptoms, nasal epithelial gene expression, and biomarkers of oxidative stress and inflammation in urine, nasal lavage fluid, and serum. They will then begin the alternate dietary intervention (MED DIET or control), depending on which they received first, and again have assessment of outcome measures at 2 and 4 weeks. As this is a feasibility study, we will assess the process and procedures necessary to perform a dietary intervention in a study population of adults with asthma.

**Implications**: Findings from this study will provide insight as to the feasibility of conducting a clinical trial of a dietary intervention in a population of adults with asthma. The results may provide preliminary evidence of the effect of a Mediterranean-like dietary intervention on lung function, inflammation, oxidative stress, and symptoms in asthma and provide insight into the duration of the intervention and the sample size needed to conduct a definitive trial.

1. **Objectives**

The primary objective of this study is to determine the feasibility of a randomized, crossover trial of a Mediterranean-like dietary intervention compared to a usual diet on lung function, oxidative stress, inflammation and symptoms in asthma. The completion of this study will inform the design for a larger trial.

1. **Background**

A Mediterranean-like diet has been proposed as a “respiratory healthy” diet. The traditional Mediterranean diet is characterized by an increased intake of plant foods such as fruits and vegetables, bread and cereals (primarily whole grain), legumes and nuts, with olive oil serving as the principal fat source. It is rich in both antioxidants and cis-monounsaturated fatty acids and provides an important source of dietary antioxidants. Adherence to the Mediterranean diet has been shown to be protective for clinically significant asthma and allergic disease in multiple cross-sectional observational studies. In a cross-sectional survey of 1,784 pre-school aged children, adherence with the Mediterranean diet was a protective factor for current wheezing.^1^ Similarly, in a cross-sectional observational study of 690 children aged 7-18 years, adherence to a traditional Mediterranean diet was associated with reduced incidence of allergic rhinitis and a trend towards decreased incidence of wheezing ^2^ In a study of 174 asthmatics with a mean age of 40 years, high adherence to the Mediterranean diet had a 78% reduction in the risk of having uncontrolled asthma (defined as FEV_1_ >80% predicted, exhaled nitric oxide >35 ppb or asthma control questionnaire score > 1.0) after controlling for gender, age, education, inhaled corticosteroid use and energy intake (OR = 0.22; 95% CI = 0.05-0.85;p=0.028). ^3^ Recent studies that have measured adherence to a traditional Mediterranean diet have shown better control of asthma in adults and improvement in asthma symptoms and rhinitis in children.^4^ As research focuses on favorable dietary patterns, a countering question is whether there exists an unfavorable diet that worsens asthma, specifically in susceptible minority populations. An observational study conducted in ~56,000 females in France showed that a dietary pattern loaded heavily with pizza/salty pies, dessert, cured meats and pasta, representing a diet high in fat, simple sugars and carbohydrates was associated with asthma severity.^5^ Specifically, this “western diet”, a diet similar to that common in inner city Baltimore African-Americans, was associated with increased risk of reporting frequent asthma attacks, whereas the “nuts and wine” diet, which was loaded heavily with nuts and seeds, olives, wines and fortified wine, was associated with a decreased risk of reporting frequent asthma attacks. To date, associations between asthma health and dietary intake have largely been investigated using observational studies.

The current study is designed to assess the feasibility of a large-scale dietary intervention trial comparing a Mediterranean-like diet to a Western-style diet. The dietary intervention includes a Mediterranean-like diet that approximates that from one of the three arms (the diet is rich in unsaturated fats) studied in the Optimal Macro-Nutrient Intake Health Trial to Prevent Heart Disease (OMNI-Heart) Trial ^6^. We will compare this to a control diet that represents the participant’s usual diet.

1. **Study Procedures**
2. **Study Design Summary**


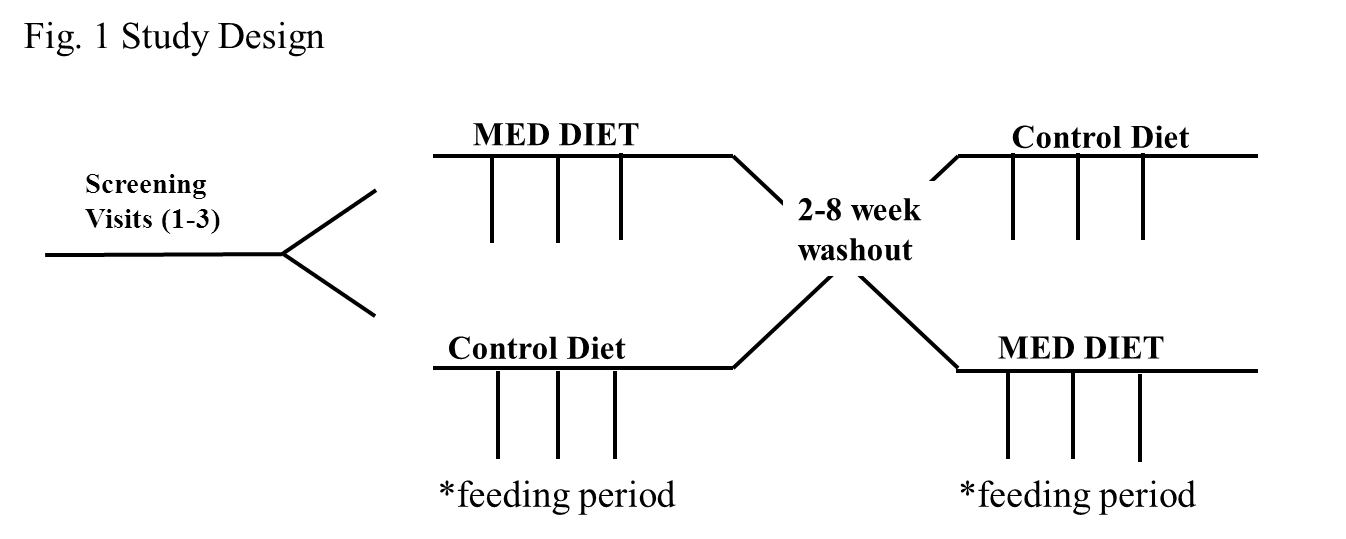

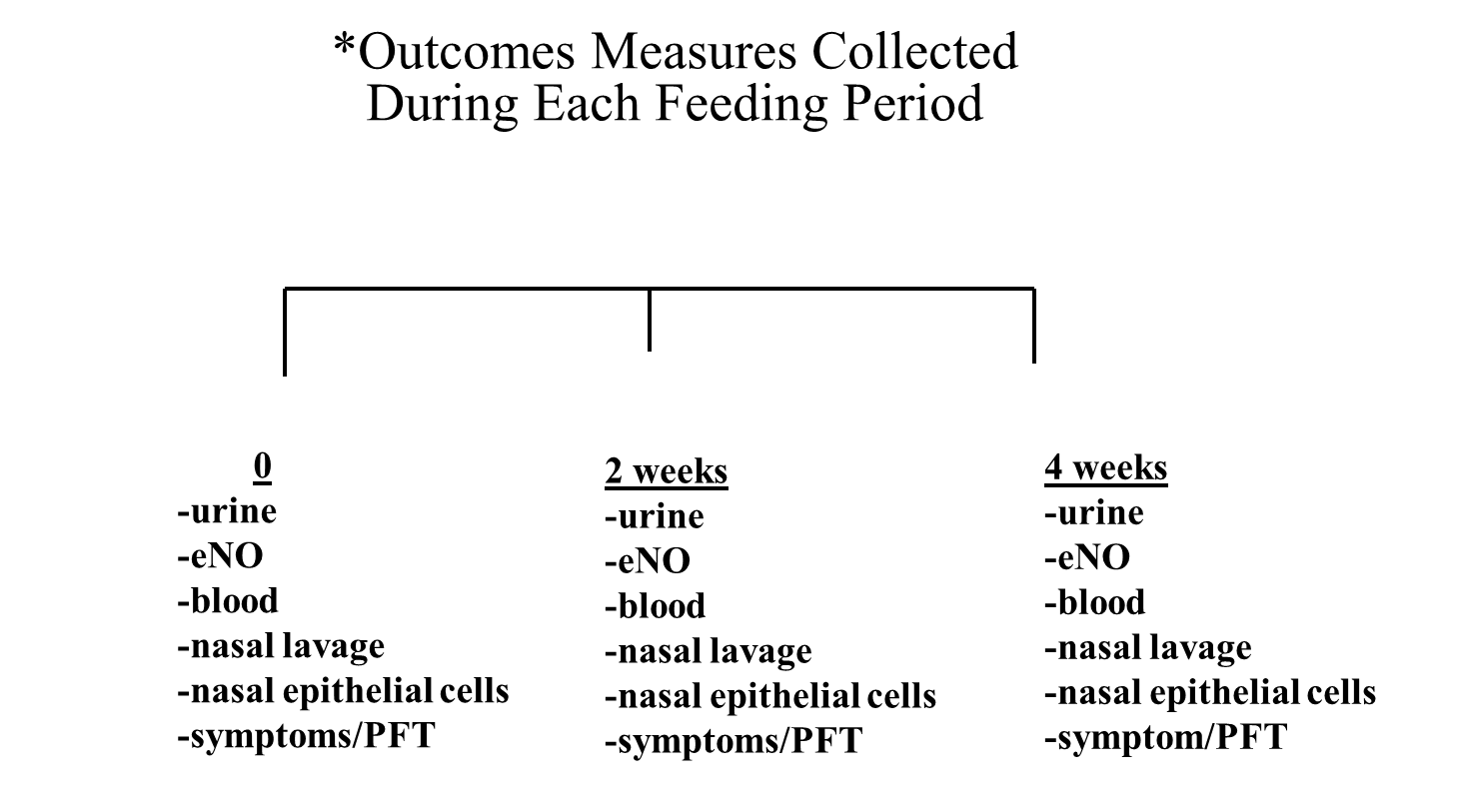


*Randomized, crossover trial*

Potential study subjects will undergo screening for eligibility at 2-3 clinic screening visits. At screening visit 1, they will have urine cotinine and questionnaires. At screening visit 2, they will complete questionnaires. At screening visit 3, they will be randomized to the MED DIET or control diet, defined as their regular diet (Figure 1). FENO, lung function, nasal epithelial gene expression, urinary oxidative stress (OS) biomarkers, serum inflammatory and OS biomarkers, and symptoms will be assessed at clinic visits before, 2 weeks after, and 4 weeks after starting each dietary intervention. Participants will then have a 2-8 week washout period during which they will resume their regular diet before continuing with the alternate dietary intervention. If the participant began on the controlled diet the washout may be shorten to 2 weeks as needed for logistical purposes.

**b. Recruitment**

The primary recruiting tool will be mass mailing of flyers and letters with follow-up telephone calls. This approach has been used in our current and completed asthma studies. Mailing will include letters in which participants will be given the choice to opt out from being further contacted and participants who do not opt out may receive a follow-up telephone call. Potential participants will be identified though research databases [NA_00026977: Effect of Fenzian treatment on symptoms, pulmonary function and Albuterol/Salbutamol use in patients with mild to moderate persistent asthma. A multicenter SHAM controlled clinical trial; screen failures from NA_00035701: Spiromics that expressed an interest in being contacted for future studies; NA_00033424: Recruitment Database of Volunteers for Research Studies in the Division of Allergy and Clinical Immunology; NA_000006137: Future Subject Database].

The IRB approved flyer will be passed out at community events (ie: health fairs, markets and fairs) and throughout the neighborhood community (mall kiosks, bus stops).

We will also recruit patients from the Principal Investigator’s and Co-Investigator’s Pulmonary Clinics who have agreed to be contacted about research studies. In addition, we will also recruit potential subjects who are referred from other physicians and have given permission to be contacted. These potential participants will not be approached by research staff or investigators unless they have given permission to their doctors and this permission has been documented in the medical record. All such potential subjects will be given information about the study by their doctor along with study team contact information. They may initiate contact with the study team unless they have given permission to be contacted by a study team member and it is documented in their medical record.

Patients from Johns Hopkins and Johns Hopkins Community Physicians Clinics will also be a source of recruitment and will receive letters with follow-up telephone calls in which patients will be given the choice the opt out from the being contacted and patients who do not opt out may receive a follow-up telephone call. We will recruit at community events. This approach has been successfully used in previous asthma studies.

**c.Eligibility criteria**

The following inclusion/exclusion criteria that must be met at screening visits to proceed with further study activities are:

**Inclusion Criteria:**

- Diagnosis of asthma, defined as
  - Physician diagnosis of asthma

and

- - Current treatment for asthma by a healthcare provider within the preceding twelve months. [Current asthma treatment defined as regular use of asthma medications. Asthma medications include short and long acting adrenergic bronchodilators, bronchodilator combinations, inhaled anticholinergics, inhaled corticosteroids, cromolyn sodium and nedocromil, leukotriene modifiers and methylxanthines]
- Stable asthma, defined by no asthma exacerbation (ED visit, course of increased systemic steroids, or urgent health care visit for asthma) during the prior four weeks.
- Non-smoker, defined by no cigarettes in the past 1 month and a negative urine cotinine
- Not breastfeeding
- Age 18-50 years
- No other major pulmonary disease such as cystic fibrosis or COPD
- Willing to eat at least one on-site meal/day, 3-4 days a week, and willing to eat study diets and nothing else for the 4 weeks of controlled feeding

**Exclusion Criteria:**

- Chronic oral steroid therapy (daily)
- Oral corticosteroid use within past 4 weeks
- Respiratory tract infection within past 4 weeks
- Significant medical issues such as heart disease or poorly controlled hypertension, type 1 diabetes, poorly controlled type 2 diabetes, or hypothyroidism that would interfere with collection of outcome measures or present safety issues in the opinion of the Principal Investigator
- Pregnancy (self-report), planning a pregnancy, or nursing/breastfeeding mothers
- Food allergy
- Consumption of more than 14 alcoholic drinks per week, or consumption of 6 or more drinks on an occasion, one or more occasions per week
- Food preferences, intolerances, or dietary requirements that would interfere with diet adherence
- Weight over 350 pounds
- Weight loss or gain of 10 pounds or more during prior 2 months
- Any condition or compliance issue which in the opinion of the investigator might interfere with participation in the study
- Use of warfarin

**d.Participant eligibility visits**

Participant eligibility is determined by an initial telephone or in-person pre-screening process and in a series of 2-3 formal screening visits, each of which includes questionnaires and clinical measurements. Data collected in the screening visits also provide baseline levels used to describe participants.

**Pre‑Screen Contact:** This contact is conducted over the phone or in-person. The pre-screening form includes brief questions on major eligibility criteria. Eligible and interested individuals from the pre-screen contact are scheduled for the first formal screening visit.

**Screening Visit 1 (SV1):** The study protocol and eligibility criteria will be reviewed with the participant. If the participant appears to be potentially eligible and willing to participate, the informed consent process will take place. SV1 includes:

- A rapid urine cotinine test will be performed for all participants to verify non-smoking status.
- Measurement of height and weight
- A screening questionnaire will be administered by study staff and will capture socio-demographic information, pulmonary and allergic history, smoking history, medications, use of birth control for women, and review of dietary habits, food allergies, and preferences (used to assess a participant’s ability and willingness to consume all diets and to adhere to the feeding protocol).
- Asthma Control Test (ACT)

**Screening Visit 2* (SV2):** A second screening visit will be conducted to increase the likelihood of adherence to the study protocol and will include collection of baseline data. Participants will receive a tour of the feeding site and an orientation to the study protocol. SV2 includes:

- Asthma health questionnaires
- Tour of facility and orientation session
- Caloric questionnaire

**Screening Visit 3*(SV3):** A third screening visit will be conducted to increase the likelihood of adherence to the study protocol and will include collection of baseline data. Participants will receive study protocol of procedures brochure. SV3 includes:

- Pre and post-bronchodilator spirometry
- Collection of blood, urine, nasal lavage, exhaled nitric oxide testing and nasal epithelial cells
- Randomization

*In some cases SV2 and SV3 maybe combined for logistical considerations.

**Randomization:** Participants will be randomized at SV3. Participants will be aware of their assignment as the study cannot be blinded.

**e.Intervention**

Description of Diets

The nutrient targets of the MED DIET intervention in current study will be based on those from the unsaturated fat diet based on an intervention arm used in Omni heart. After randomization, participants are either continued on their usual diet or fed the intervention diet. The MED DIET is low in saturated fat and cholesterol, emphasizing unsaturated fat (21% of kcal), predominantly monounsaturated fat. This diet uses nuts, seeds, and oils such as olive, canola, and safflower oil to meet its target fat distributions. Specially formulated fat products are not used. For each diet, five calorie levels are prepared (1600, 2000, 2500, 3000 and 3500 kcal). The macronutrient profile is identical at each calorie level. The micronutrient levels are based on a Linear Index Model that indexes micronutrient levels to energy levels ^7^. This model is based on actual population consumption data and thus provides a realistic range of micronutrient intakes at lower and higher calorie levels, rather than a fixed ratio applied to all calorie levels (e.g. 4,700 mg of K per 2,100 kcal, or 2,238 mg of K per 1,000 kcal). Participants will be assigned a calorie level based on questionnaire administered at SV2. The calorie level will be adjusted as needed during the study protocol to maintain the baseline weight.

## Food Production and Distribution

Food production and distribution will occur at the ProHealth Clinical Research Unit using procedures that have been successfully employed in previous studies ^6-8^ Once menus are formulated, all foods are identified and selected to promote consistency. Specific national brands are selected, as well as purchasing specifications for meats and produce. Food production is also conducted according to respective state or county public health guidelines and JCAHO regulations. Quality control procedures developed in the DASH trials are used to monitor food procurement, preparation, and distribution. Research kitchens are monitored for safety, sanitation and equipment accuracy.

The process of preparing foods is a labor intensive process. In this process, food service workers weigh, portion, and package individual food items for the three diets, according to the calorie levels of the participants being fed. The cooks prepare all cooked items (e.g. casseroles, meats); in the process, they measure raw product, cook the items following standard hygienic procedures, and store the individualized portions. Some portion-controlled packaged food items are used for production efficiency. A diet technician assembles the trays for on-site meals and the bags and coolers for distribution of off-site meals, by diet and calorie level. A second person independently rechecks the assembled foods to confirm the accuracy of food delivery.

The feeding protocol is very similar to that used in the DASH and DASH-Sodium trials. All study food is provided to participants who are instructed to eat all their food and to consume no additional food other than approved selected beverages. Participants are required to eat one meal (the main meal of the day) at the feeding center for 4 days a week for the first 2 weeks of the 4 week MED DIET intervention. The remaining food for the day and weekend food are provided for consumption off-site. During the third, and fourth weeks of the MED DIET, participants will eat 1 main meal on-site for three days a week and will be provided with the remainder of their food for consumption off-site. On any given feeding visit, participants are expected to complete a daily food diary and be weighed. The daily diary asks about study foods not eaten, non-study foods eaten, and beverages consumed over the past day.

A dietitian case manager meets participants at the feeding center, receives and reviews their progress, elicits general feedback, tracks weight, and adjusts the calorie level, if needed. The participant then proceeds to the dining area to eat the on-site meal. A meal monitor checks the completed tray and provides the take-home meals. On each day of feeding, participants complete a daily diary, which elicits information about food and beverage consumption over the preceding day, including non-alcohol beverages consumed, alcohol beverages consumed, unit foods eaten, study foods not eaten, non-study foods eaten, and vitamin/supplement consumed.

## Promotion of Adherence

Efforts to promote adherence begin at the earliest stages of the study. During screening and orientation, participants are repeatedly provided with information about key features of the study. At the screening visit, they are provided a detailed list of foods provided in the diets. Individuals must be willing to eat each of these foods; otherwise, they are excluded. Key contacts with dietary staff include an in-person evaluation by a dietitian. The intent of these efforts is to identify and exclude, prior to randomization, participants who are unwilling or unable to comply with the feeding protocol.

Efforts to promote adherence center on making the foods palatable and convenient to the participant lifestyles; maintaining easy access to staff; and providing daily, supportive contacts. Acceptance of the controlled feeding protocol is increased by allowing participants to consume pre- approved selected beverages, as well as an unlimited amount of water and artificially sweetened soft drinks.

**Assessment of Adherence**

Adherence assessment includes both self-reported and objective measures. The subjective measures are used to determine suitability for randomization and subsequently to counsel participants and promote adherence during the trial. Self-reported measures are obtained from information provided on a daily diary and from subjective judgment of clinic personnel. Each day, an overall compliance score (0=compliant; 1,2, or 3 for various degrees of non-compliance) is calculated based on staff observation and information from the daily diary. The objective measures, available at the end of the study, are used to document the overall success of our procedures. Objective measures include serum carotenoids, meal attendance and on-site meal consumption; and body weight.

### Intervention Periods

Each of two feeding periods lasts 4 weeks. During the MED DIET intervention period, participants are provided all of their food, snacks and most beverages. For each day of this controlled feeding, participants also complete a daily diary. During the last week of the last feeding period, participants complete an anonymous adherence questionnaire. During the control diet period, participants will consume their regular diet. A washout period of at least 2-8 weeks separates each period; the washout period allows ad libitum food intake. Participants are fed sufficient calories to maintain their weight. Participants have serum, urine, ENO, nasal lavage, nasal cells collected and questionnaires completed before and at approximately 2 and 4 weeks after each intervention. Table 2 displays major measurements and data collection activities by visit. The following sections describe our approach to measuring all outcomes variables and other selected measurements.

**f.Study Procedure Methods**

**Intervention**

During the MED DIET intervention, participants are provided all of their food, snacks and most beverages. On weekdays, participants will eat some of their major meals on-site, either lunch or dinner, and receive the remainder of their meals to be eaten off-site. For weekend meals, they are provided all of their food on the preceding Thursday or Friday. For each day of controlled feeding, participants complete a daily diary which asks about study food not eaten, non-study food eaten, the number of caffeinated beverages consumed, and the number of alcohol beverages consumed.

**Table 1: Study Data Collection Table**

**Pre-**

**screening call**

**Screening**

**visit 1**

**Screening**

**visit 2**

**Screening**

**visit 3**

**Washout**

**Visit**

**SV1**

**SV2**

**SV3**

**IV1.1**

**IV1.2**

**IV2.3**

**IV2.1**

**IV2.2**

**IV2.3**

**Time (weeks)***

these times may

vary

**1**

**7-16**

**Consent/**

**eligibility**

**Randomization**

**X***

**X***

**Tour/**

**Information**

**Session**

**Asthma**

**Questionnaires**

**Urine cotinine**

**X**

**Urine**

**X***

**X***

**X**

**X**

**X**

**X**

**X**

**FENO**

**X***

**X***

**X**

**X**

**X**

**X**

**X**

**Blood Draw**

**X***

**X***

**X**

**X**

**X**

**X**

**X**

**Nasal Lavage**

**X***

**X***

**X**

**X**

**X**

**X**

**X**

**Nasal**

**Epithelial Cell**

**Collection**

**PFTs**

**X***

**X***

**X**

**X**

**X**

**X**

**X**

**Environment**

**Assessment**

**Telephone- 24**

**hour diet**

**Asthma Daily**

**Diary+**

**Intervention 1**

**Intervention 2**

**X**

**1-2**

**3-6**

**17-20**

**X**

**X***

**X***

**X**

**X**

**X**

**X**

**X**

**XXX**

**XXX**

**XXX**

**X**

**X**

**X**

**X**

**X+**

**X+**

**X**

**X**

*** These data may be collected at either SV2 or SV3. If more than 2 weeks have elaspsed between the collection of baseline clinical**

**and inflammatory data that occurs at SV2 or SV3 and IV1, these procedures will be repeated at IV1.**

**+This diary will be collected daily.**

**X**

**X***

**X***

**X**

**g*.*Questionnaires:**

The following questionnaires will be administered:

**Asthma Questionnaires**

- The Asthma Control Test (ACT) is a validated questionnaire ^9^. It integrates common indicators of asthma control including cough, nocturnal awakenings, frequency of medication use, and frequency of symptoms. It is a sensitive measure of asthma control, a difference of 3 reflects a clinically meaningful difference in asthma control. The instrument is well validated and is a short questionnaire with easy to administer questions that are easily understood.
- The Asthma Symptom Utility Index (ASUI) ^10^ is a validated 2-week recall questionnaire that addresses issues of asthma control weighted by impact on functional status.
- Asthma specific quality of life is measured with the Marks Atcham Quality of Life Questionnaire (Marks AQLQ) which has been validated for individuals 15 and older. The questionnaire is designed to measure daily impact of asthma in patients’ lives.^11^
- Juniper Asthma Control Questionnaire (ACQ) will be administered at all visits. ACQ is a validated instrument that integrates common indicators of asthma control including use of bronchodilators, nocturnal symptoms, cough, activity level, and pulmonary function.^12^  The questionnaire takes less than 5 minutes to complete.

**Asthma Daily Diary**

Participants will complete an asthma diary during the study period.^13^ Participants will complete an asthma diary during the study period that quantifies daytime and nocturnal asthma symptoms and has been validated for use as an outcome measure in clinical trials.

**Environmental Assessment**

Participants will complete an environmental assessment questionnaire that has been successfully used in our previous environmental epidemiologic studies in Baltimore at IV1.2 and IV2.2.

**24 Hour Dietary Recall**

24 hour diet recall surveys will be administered by telephone by research staff from the Penn State Diet Assessment Center. Participants will have a goal of three 24 hour dietary recall surveys administered at baseline and during each intervention period, for a goal of nine 24 hour dietary recalls. Each set of three recalls will include one weekend day. The participant will be contacted on 3 separate occasions via phone by interview staff at the Penn State Diet Assessment Center. The phone calls will be made on both weekdays and weekend days and will last about 20 minutes. During this call the interviewer will ask standard sets of questions on food and drinks that the participant consumed in the previous 24 hours. To help estimate the food amounts, participants will be provided with a Food Amount Booklet to reference during the phone assessments.

**Weight** is measured by trained, certified staff using a calibrated balance beam scale. Weight will be recorded during screening at the V1 visit (to determine eligibility and to estimate calorie requirements) and 3-4 times a week while on the MED DIET intervention (to adjust calorie intake in order to maintain weight).

**Height** (collected once at the V1 visit) is measured by trained staff using a stadiometer.

**Lung Function**

Spirometry will be performed at the baseline screening visit and at 5 follow up visits (see Table 1). Spirometry will be performed according to ATS guidelines to obtain FEV_1_, FVC, FEF_25-75_, and PEF. ^16-17^ After spirometry is obtained, participants will be administered 2 puffs of albuterol via a spacer. After 15-45 minutes, spirometry will be repeated.

**Urine**

Urine will be collected at the baseline screening visit and as a part of the 5 follow up visits (see Table 1). At the baseline screening visit, it will be collected for rapid urine cotinine measurement on all participants. Rapid urine cotinine testing will be performed with NicAlert test strips and a NicAlert of 4 or greater will exclude a participant from proceeding. Urinary biomarkers will also be measured to assess oxidative stress and inflammation at baseline and at the additional 5 follow up visits. LTE_4_ and F-isoprostane have emerged as robust markers of oxidative stress and inflammation ^10^ and will be quantified by ELISA in Dr. Biswal’s or Dr. Wills-Karp’s laboratories or by an outside commercial laboratory, along with other markers related to asthma and allergy.

**Blood**

At baseline, blood will be analyzed for allergen specific IgE levels to cockroach, mouse, cat, dog, dust mites, orchard grass, and ragweed using the ImmunoCAP system (Pharmacia Diagnostics, Uppsala, Sweden).

Blood will be collected at the screening visit 2 and on each of the 5 follow up visits for the following:

1. Serum inflammatory markers and oxidative stress markers, such as IL-5, IL-13, RANTES, superoxide anion, lipid peroxides
2. Basophil activation
3. White blood cell superoxide anion production
4. Complete blood count with differential
5. Comprehensive metabolic panel with lipids and iron
6. Serum carotenoids

Cytokines/chemokines and markers of oxidative stress will be measured in the Biswal Laboratory or the Wills-Karp Laboratory at the Johns Hopkins School of Public Health. Basophil activation will be measured in the Shreffler Laboratory at Mass General Hospital.

**h.Biospecimens and Biomarkers**

Biomarkers will be measured in the following biospecimens: nasal lavage, nasal epithelial cells, serum, blood, urine, and FENO. These will include various markers of inflammation and oxidative stress (see below).

**FENO:** Exhaled nitric oxide is a known marker of pulmonary inflammation and will provide a means of assessing pulmonary oxidative stress and inflammation. FENO will be measured during each of the 6 clinic visits. Measurement of exhaled nitric oxide will be obtained according to the American Thoracic Society Guidelines.^17^ and prior to lung function whenever possible. Nitric oxide concentrations will be measured using a chemiluminescent analyzer (NIOX Mino, Aerocrine, Sweden). This equipment is FDA-approved for clinical use in asthma management.

**Nasal Epithelial Cell Collection:** Nasal epithelial cells will be obtained during each clinic visit. The sampling will be done with the Rhinoprobe, a commercially available, disposable curette designed for such sampling, and the cell sample will be used to measure Phase II enzyme gene expression.  The nasal cavity will be held open using an autoclaved nasal speculum.  The curette will be inserted into the nose under direct visual inspection.  Gentle pressure will be applied to the curette as it is drawn gently along the inferior surface of the inferior nasal turbinate 10 times.  The curette will then be placed in an eppendorf tube containing lysis buffer.  RNA will be extracted for analysis of gene expression using RT-qPCR, such as the phase II enzymes, GCLM, GCLC, HO-1, as well as expression of other genes related to asthma or allergy.

**Nasal Lavage Procedure:** Nasal lavage will be performed at each clinic visit. To undergo nasal lavage, a participant will be instructed to inspire deeply and hold his or her breath so that the soft palate seals off the nasopharynx. The participant will then extend his or her neck backwards, at a 45 degree angle, while the lavage solution is instilled in the two nostrils with the use of a pipette. The lavage solution dwells in the nasal passages for 10 seconds, while the study participant holds his or her breath. Next, the participant will be instructed to lean forward and expel the lavage fluid into an emesis basin. The return fluids will be shaken and kept on ice until they are centrifuged. Following centrifugation, pellets from the lavages will be used for cytology. The supernatants of lavages will be used for analysis of mediators and cytokines.

**Analysis of Biomarkers in Nasal Specimens:** The following biomarkers/correlative assays will be run on nasal lavage specimens in either our laboratory, the Biswal laboratory in the JHSPH: (1) cell count and differential, (2) cytokines and chemokines. The percentage of eosinophils, neutrophils, and mononuclear cells in nasal lavage fluid will be quantified. The supernatant will be stored frozen until assayed for cytokines, chemokines, and other markers of inflammation. Cytokines/chemokines will be measured by ELISA using multi-plex antibody bead kits from Lincoplex (St. Charles, Missouri) according to the manufacturer’s instructions or other comparable immunoassay. Tryptase levels will be quantified by fluoroenzyme immunoassay in the Matsui laboratory (ImmunoCAP Tryptase, Pharmacia Diagnostics, Uppsala, Sweden).

**i.Blinding, including justification**

It is not possible to blind participants to this protocol as they will consume the intervention diet at the feeding center and the control diet will consist of their usual diet. Participants will be randomized using a computerized randomization scheme to receive either the MED DIET or the control diet first and they will receive the alternate diet after the washout period. The nutrition core at ProHealth will dispense the dietary interventions to the study participants. The study staffs who will have access to the randomization scheme are Roger Peng, PhD (co-investigator and biostatistician) and the database programmer from the Data Core and the staff in the Nutrition Core at ProHealth.

**j.Justification why participants will not receive routine care or will have current therapy stopped**

The study protocol will not interfere with participants receiving their previous routine medical care, and asthma care. A copy of all lab (blood) results along with a copy of all pulmonary function test results obtained during their visits will be given to the participants at the end of the study accompanied by a letter for their PCP.

**k.Justification for inclusion of a placebo or non-treatment group**

Each participant will receive the intervention and the control diet in this crossover study. The participant’s regular diet will serve as the control to provide a comparison which represents the diet typically consumed by the individual.

**l.Definition of treatment failure or participant removal criteria**

We do not anticipate that participants will experience any significant side effects from ingestion of the diets.

At any time, the subject’s participation will stop if he/she requests not to proceed with the study.

**m.Description of what happens to participants receiving therapy when study ends or if a participant’s participation in the study ends prematurely**

Participants will resume their typical diet once the study concludes or when the participant stops the study.

1. **Inclusion/Exclusion Criteria**

**Inclusion Criteria:**

- Diagnosis of asthma, defined as
  - Physician diagnosis of asthma

and

- - Current treatment for asthma by a healthcare provider within the preceding twelve months. [Current asthma treatment defined as regular use of asthma medications. Asthma medications include short and long acting adrenergic bronchodilators, bronchodilator combinations, inhaled anticholinergics, inhaled corticosteroids, cromolyn sodium and nedocromil, leukotriene modifiers and methylxanthines]
- Stable asthma, defined by no asthma exacerbation (ED visit, course of increased systemic steroids, or urgent health care visit for asthma) during the prior four weeks.
- Non-smoker, defined by no cigarettes in the past 1 month and a negative urine cotinine
- Not breastfeeding
- Age 18-50 years
- No other major pulmonary disease such as cystic fibrosis or COPD
- Willing to eat at least one on-site meal a day, 3-4 days a week, and willing to eat study diets and nothing else for the 4 weeks of controlled feeding

**Exclusion Criteria:**

- Chronic oral steroid therapy (daily)
- Oral corticosteroid use within past 4 weeks
- Respiratory tract infection within past 4 weeks
- Significant medical issues such as heart disease or poorly controlled hypertension, type 1 diabetes, poorly controlled type 2 diabetes, or hypothyroidism that would interfere with collection of outcome measures or present safety issues in the opinion of the Principal Investigator
- Pregnancy( self-report), planning a pregnancy, or nursing/breastfeeding mothers
- Food allergy
- Consumption of more than 14 alcoholic drinks per week, or consumption of 6 or more drinks on an occasion, one or more occasions per week
- Food preferences, intolerances, or dietary requirements that would interfere with diet adherence
- Weight over 350 pounds
- Weight loss or gain of 10 pounds or more during prior 2 months
- Any condition or compliance issue which in the opinion of the investigator might interfere with participation in the study
- Use of warfarin

1. **Drugs/Substances/Devices**
2. **Study Statistics**

**a.Primary outcome variable:**

As this is a pilot study, our outcomes of interest are related to process and procedures necessary to successfully deliver a dietary intervention to a population of adults with asthma. We have chosen a convenience sample of approximately 12 participants for this feasibility study. To assess feasibility, we will assess numbers of participants screened for each enrolled, reasons for screen fails, rate of enrollment, and numbers of drop-outs. We will assess adherence to the Mediterranean dietary intervention using both self-reported and objective measures. Self-reported measures are obtained from information provided on a daily diary and from subjective judgment of clinic personnel. Each day, an overall compliance score (0=compliant; 1, 2, or 3 for various degrees of non-compliance) is calculated based on staff observation and information from the daily diary. The objective measures include serum carotenoid values, meal attendance, on-site meal consumption; and body weight. We will measure clinical and biological endpoints to inform the design of a larger, more definitive trial, including asthma symptoms scores and lung function.

**b.Early stopping rules**

As this is a pilot study there will be no formal criteria for stopping the study early, and there is no plan for interim analysis for individuals. However participants may choose leave the study early or at the determination of the Principal Investigator.

1. **Risks**

**a.Medical risks, listing all procedures, their major and minor risks and expected frequency**

Questionnaires: The principal risk associated with questionnaires is loss of confidentiality.

Spirometry: The minor risk is the slight discomfort of forceful exhalation.

The drug albuterol can lead to tremor, nervousness, tachycardia, palpitations, and headache. These reactions are transient and rare (< 5%) with the proposed doses used for this study, and if they occur, we will monitor the patient until they return to baseline. High dose albuterol may cause arrhythmias and hypokalemia. However, these reactions are very unlikely with the doses used for this study.

Venipuncture: There is a risk of minor discomfort or bruising at the site of the blood draw. In addition, some people experience lightheadedness. Very rarely, an infection can develop at the venipuncture site.

Nasal cell collection: A risk of cell sampling is mild discomfort while the sample is being collected; this feeling should go away immediately after the sample is taken. There is a slight risk of some nasal bleeding during or just after the sample is collected. Rarely an infection may occur at the sampling site

Nasal Lavage: Minimal discomfort and a small risk of aspiration of the lavage fluid.

Dietary intervention (MED DIET) The diet interventions pose minimal risk. Participants may experience some bloating and other minor gastrointestinal discomforts related to the high fruit, dairy and fiber content of the intervention diets. It has been our experience that these problems resolve soon after changes in diet. For those persons with lactose intolerance, we provide lactaid. Our experience suggests that GI discomfort is generally minor and subsides quickly. Participants are monitored for reactions to the diets and, if necessary, the diet can be modified or terminated*.*

**b.Steps taken to minimize the risks**

Questionnaires: Unique identifiers will be assigned to participants and these will be used to label all paperwork, including questionnaires.

Spirometry: Will be performed by trained study personnel.

Venipuncture: A staff member who is experienced in phlebotomy will perform venipuncture.

Nasal cell collection: A staff member experienced and trained in nasal epithelial cell collection will perform the procedure.

Nasal Lavage: A staff member experienced and trained in nasal lavage will perform the procedure. To reduce the risk of aspiration a small volume of lactated ringers 10 ml (5 ml per nostril) will be instilled into the nasal cavity and subjects will be instructed to hold their breath to seal off the nasopharynx.

Dietary intervention (MED DIET**):** GI discomfort is generally minor and subsides quickly. Participants are monitored for reactions to the diets and, if necessary, the diet can be modified or terminated.

**c.Plan for reporting unanticipated problems or study deviations**

Any serious or unexpected adverse event will be reported to the IRB according to institutional reporting requirements; other adverse events will be reported to the IRB at the time of continuing review. Protocol deviations will be reported to the IRB according to institutional reporting requirements: minor or administrative deviations to the protocol will be reported with the continuing review; major, non-emergent deviations will be submitted for review; and emergent deviations will be reported as soon as possible and at least within 5 days to the IRB.

**d.Legal risks such as the risks that would be associated with breach of confidentiality**

The risk of breach of confidentiality will be minimized by using unique identifiers for participants and keeping the record that links the identifier to the participant in a locked file cabinet or office and/or password protected database accessible only to the investigators and study staff.

**e.Financial risks to the participants**

In the rare event that a participant has a reaction to diet intervention and he/she seeks medical care, he/she will be responsible for payment of those services. All costs associated with diet intervention, blood drawing, questionnaires and spirometry are covered by the investigators**.**

1. **Benefits**

The results of the study will lend important insight into the role of diet in asthma clinical symptoms, lung function, and inflammation and oxidative stress. The study may also provide insight as to the potential for treating asthma with dietary interventions.

1. **Payment and Remuneration**

A study participant will receive compensation according to the table below. In addition, participants will be reimbursed for any transportation fees that are needed in extenuating circumstances for travel to and from the ProHealth Site for study purposes. They will also receive all of their meals during the MED DIET intervention periods. Participants will receive an insulated bag to transport study food to and from ProHealth and will be able to keep bag at the end of the study.

| **Study Activity/Procedure** | **Compensation** |
| --- | --- |
|  |  |
| Completion of dietary intervention and associated study procedures | $150/intervention period x 2 intervention periods = $300  100 |
|  |  |
| Telephone completion of 24 hour diet  recall (9) | $180 |
| Bonus for completing entire study | $100 |
|  |  |
| **Total Possible Compensation** | **$580** |
|  |  |

1. **Costs**

All costs that are associated with the study procedures are covered by the investigators.

**Bibliography**

1. Castro-Rodriguez JA, Garcia-Marcos L, fonseda Rojas JD, Valverde-Molina J, Sanchez-Solis M. Mediterranean diet as a protective factor for wheezing in preschool children. *J Pediatr* 2008 June;152(6):823-8, 828.
2. Chatzi L, Apostolaki G, Bibakis I et al. Protective effect of fruits, vegetables and the Mediterranean diet on asthma and allergies among children in Crete. *Thorax* 2007 August;62(8):677-83.
3. Barros R, Moreira A, Fonseca J et al. Adherence to the Mediterranean diet and fresh fruit intake are associated with improved asthma control. *Allergy* 2008 July;63(7):917-23.
4. de BJ, Garcia-Aymerich J, Barraza-Villarreal A, Anto JM, Romieu I. Mediterranean diet is associated with reduced asthma and rhinitis in Mexican children. *Allergy* 2008 October;63(10):1310-6.
5. Varraso R, Kauffmann F, Leynaert B et al. Dietary patterns and asthma in the E3N study. *Eur Respir J* 2009 January;33(1):33-41.
6. Appel LJ, Sacks FM, Carey V et al for the OMNI-Heart Collaborative Research Group The Effects of Protein, Monounsaturated Fat, and Carbohydrate Intake on Blood Pressure and Serum Lipids: Results of the OmniHeart Randomized Trial. JAMA, 2005; 294:2455-64.
7. Lin P, Aickin M, Champagne C, et al. Food group sources of nutrients in the dietary patterns of the DASH-sodium trial. J Am Diet Assoc 2003; 103:488-496.
8. Carey VJ, Bishop L, Charleston J, et al. Rationale and design of the optimat macro-nutrient intake heart trial to prevent heart disease (OMNI-Heart). Clinical Trials 2005; 2:529-537.
9. Schatz M, SorknessCA, Li JT,Marcus P, et al. Asthma Control Test: reliability, validity, and responsiveness in patients not previously followed by asthma specialists. J Allergy Clin Immunol 2006;117:549– 55
10. Revicki DA, Leidy NK, Brennan-Diemer F, Sorensen S, Togias A:Integrating patient preferences into health outcomes assessment: the multiattribute Asthma Symptom Utility Index. Chest 1998, 114:998-1007.
11. Juniper, E.F., et al., Development and validation of a questionnaire to measure asthma control. Eur Respir J, 1999. **14**(4): p. 902-7.
12. Juniper EF. Health-related quality of life in asthma. *Curr Opin Pulm Med* 1999;5:105-110.
13. Santello N.C, Barber B.L. et al. Measurement characteristics of two asthma symptom diary scales for use in clinical trial 1997: 650
14. Hankinson JL, Odencrantz JR, Fedan KB. Spirometric reference values from a sample of the general U.S. population. *Am J Respir Crit Care Med* 1999;159:179-187.
15. Miller MR, Hankinson J, Brusasco V et al. Standardisation of spirometry. *Eur Respir J* 2005;26:319-338
16. Dworski R, Roberts LJ, Murray JJ, et al. Assessment of oxidant stress in allergic asthma by measurement of the major urinary metabolite of F2-isoprostane, 15-F2t-IsoP (8-iso-PGF2alpha). Clin Exp Allergy 2001; 31(3):387-90.
17. American Thoracic Society/European Respiratory Society. ATS/ERS recommendations for standardized procedures for the online and offline measurement of exhaled lower respiratory nitric oxide and

nasal nitric oxide, 2005. Am J Respir Crit Care Med 2005;171:912–930.
